# Diabetes and the risk of cardiovascular events and all‐cause mortality among older adults: an individual participant data analysis of five prospective studies

**DOI:** 10.1111/eci.14340

**Published:** 2024-10-28

**Authors:** Valerie Aponte Ribero, Orestis Efthimiou, Nazanin Abolhassani, Heba Alwan, Douglas C. Bauer, Séverine Henrard, Antoine Christiaens, Denis O’Mahony, Wilma Knol, Mike J. L. Peters, Arnaud Chiolero, Drahomir Aujesky, Gérard Waeber, Nicolas Rodondi, Cinzia Del Giovane, Baris Gencer

**Affiliations:** ^1^ Institute of Primary Health Care (BIHAM) University of Bern Bern Switzerland; ^2^ Graduate School for Health Sciences University of Bern Bern Switzerland; ^3^ Institute of Social and Preventive Medicine University of Bern Bern Switzerland; ^4^ Department of Epidemiology and Health Systems, Center for Primary Care and Public Health (Unisanté) University of Lausanne Lausanne Switzerland; ^5^ Department of Medicine and Epidemiology & Biostatistics University of California San Francisco San Francisco California USA; ^6^ Clinical Pharmacy research group, Louvain Drug Research Institute (LDRI) Université catholique de Louvain Brussels Belgium; ^7^ Institute of Health and Society (IRSS) Université catholique de Louvain Brussels Belgium; ^8^ Fonds de la Recherche Scientifique – FNRS Brussels Belgium; ^9^ School of Medicine University College Cork Cork Ireland; ^10^ Department of Geriatric Medicine and Expertise Centre Pharmacotherapy in Old Persons University Medical Center Utrecht, Utrecht University Utrecht Netherlands; ^11^ Population Health Laboratory (#PopHealthLab), Department of Community Health University of Fribourg Fribourg Switzerland; ^12^ School of Population and Global Health McGill University Montreal Canada; ^13^ Department of General Internal Medicine Inselspital, Bern University Hospital, University of Bern Bern Switzerland; ^14^ Department of Medicine Lausanne University Hospital (CHUV), University of Lausanne Lausanne Switzerland; ^15^ Department of Medical and Surgical Sciences for Children and Adults University‐Hospital of Modena and Reggio Emilia Modena Italy; ^16^ Cardiology Division Geneva University Hospitals Geneva Switzerland; ^17^ Service of Cardiology Lausanne University Hospitals Lausanne Switzerland

**Keywords:** all‐cause mortality, cardiovascular disease, coronary heart disease, diabetes

## Abstract

**Background:**

Guidelines and studies provide conflicting information on whether type 2 diabetes (T2D) should be considered a coronary heart disease risk (CHD) equivalent in older adults.

**Methods:**

We synthesized participant‐level data on 82,723 individuals aged ≥65 years from five prospective studies in two‐stage meta‐analyses. We estimated multivariable‐adjusted hazard ratios (HRs) and 95% confidence intervals (CIs) of T2D (presence versus absence) on a primary composite outcome defined as cardiovascular events or all‐cause mortality. Secondary outcomes were the components of the composite. We evaluated CHD risk equivalence by comparing outcomes between individuals with T2D but no CHD versus CHD but no T2D.

**Results:**

The median age of participants was 71 years, 20% had T2D and 17% had CHD at baseline. A total of 29,474 participants (36%) experienced the composite outcome. Baseline T2D was associated with higher risk of cardiovascular events or all‐cause mortality versus no T2D (HR 1.44, 95% CI [1.40–1.49]). The association was weaker in individuals aged ≥75 years versus 65–74 years (HR 1.32 [1.19–1.46] vs. 1.56 [1.50–1.62]; *p*‐value for interaction = .032). Compared to individuals with CHD but no T2D, individuals with T2D but no CHD had a similar risk of the composite outcome (HR 0.95 [0.85–1.07]), but a lower risk of cardiovascular events (HR 0.76 [0.59–0.98]).

**Conclusions:**

T2D was associated with increased risk of cardiovascular events and all‐cause mortality in older adults, but T2D without CHD conferred lower risk of cardiovascular events compared to CHD without T2D. Our results suggest that T2D should not be considered a CHD risk equivalent in older adults.

## Graphical abstract

In this analysis of individual participant data of five prospective studies including 82,723 individuals aged ≥65 years, type 2 diabetes (T2D) was associated with increased risk of future cardiovascular events and all‐cause mortality compared to no T2D. However, individuals with T2D without coronary heart disease (CHD) conferred lower risk of future cardiovascular events compared to those with CHD but no T2D, suggesting that T2D should not be considered a CHD risk equivalent in older adults.

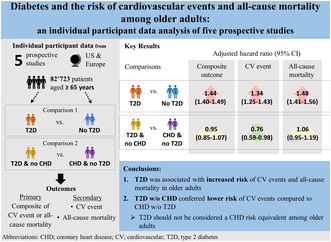

## 1 INTRODUCTION

Almost 20% of adults aged 65 years and older are estimated to be living with diabetes worldwide.^1^ Type 2 diabetes (T2D) is estimated to increase the risk for cardiovascular disease (CVD) and all‐cause mortality by a factor between 2 and 4 in the general population.^2^, ^3^ However, the strength of the association in older adults (≥65 years) is less clear. The American Heart Association, American College of Cardiology and American Geriatrics Society have highlighted the need for clinical studies that better represent the broad spectrum of older adults and address knowledge gaps in the prognosis and CVD risk stratification in the older population.^4^ In fact, existing meta‐analyses were limited to patients without established CVD and it is unclear whether the association between T2D and CVD or mortality may differ between those with and without preexisting CVD within older age groups.^2^, ^5^ Evidence on differences by gender within older age groups is also sparse. While a meta‐analysis evaluated sex‐differences in cardiovascular mortality for patients with T2D in older age groups, nonfatal CVD was not included and the population was again limited to patients without established CVD.^5^ Moreover, existing meta‐analyses are primarily based on data from the early 2000s or older while important advances in diabetes management and CVD prevention have been made since then.^2^, ^5^

In particular, it is controversial whether older adults with diabetes have a CVD risk similar to that of older adults with established coronary heart disease (CHD). While some current guidelines consider T2D to be a CHD equivalent in terms of CVD risk assessment,^6^, ^7^ evidence on whether T2D confers a similar risk of CVD as established CHD is conflicting with regards to older adults.^8^, ^9^, ^10^, ^11^ Limitations of previous studies on older adults included the exclusion of nonfatal CVD events,^9^ the absence of cause‐specific mortality data,^10^ and a focus on gender‐specific study populations.^11^ A better understanding of the association between T2D and CVD and all‐cause mortality in older adults may contribute to improved CVD risk stratification of older individuals with T2D.

Pooling individual participant data from multiple studies is one powerful strategy to address these questions as it increases statistical power over single studies, while allowing for harmonization of variable definitions and analysis methods across studies.^12^ Therefore, we pooled individual participant data from five prospective studies that were not included in previous meta‐analyses to clarify the association between T2D and CVD events and all‐cause mortality, and the status of T2D as a CHD risk equivalent, in older adults aged ≥65 years. We further aimed to evaluate these associations across subgroups of age (65–74 vs. ≥75 years), gender and presence of established CVD. Additionally, we evaluated the association between HbA1c values and CVD events and all‐cause mortality in this population.

## 2 METHODS

### 2.1 Study population

We included individual participant data from five prospective studies conducted in the United States (US) and Europe: the Cohorte Lausannoise (CoLaus) study, a Swiss population‐based cohort study of 6188 individuals aged 35–75 years of which 915 participants aged ≥65 years were included^13^; the Health, Aging, and Body Composition (Health ABC) study, a US population‐based cohort study of 3075 community‐dwelling individuals aged 70–79 years^14^; the Health and Retirement Study (HRS), a US longitudinal panel study of over 37,000 individuals aged ≥50 years of which 16,781 participants aged ≥65 years were included^15^; the Optimising Therapy to Prevent Avoidable Hospital Admissions in Multimorbid Older People (OPERAM) study, a multicentre randomized controlled trial assessing the effectiveness of a computer software in reducing drug‐related hospital admissions of 2008 participants aged ≥70 years with multimorbidity and polypharmacy in four European countries of which 1980 participants with follow‐up data were included (this study was analysed as a cohort, as done previously,^16^ that is, both randomization arms were included given that there were no differences in drug‐related hospitalizations and all‐cause mortality after 1 year)^17^; and the Survey of Health, Ageing and Retirement in Europe (SHARE), a longitudinal panel study on over 140,000 individuals aged ≥50 years in 27 European countries and Israel of which 59,972 participants aged ≥65 years were included.^18^, ^19^ Studies are briefly described in the Appendix S1. All studies received ethical approval and obtained participant's informed consent. The protocol of the current study was approved by the cantonal ethics committee in Bern, Switzerland and is available on medRxiv.^20^

For the current analysis, we included participants aged 65 years and older at baseline (see the Appendix S1 for definitions of baseline for each study). Participants with type 1 diabetes were excluded. Figures S1 and S2 present flow diagrams of participant selection.

### 2.2 Definition of exposures

Baseline T2D was defined using any of the following criteria: (i) Self‐reported diabetes diagnosis or ascertained from medical records; (ii) Self‐reported diabetes medication use or ascertained from medical records; (iii) Diabetes according to diagnostic criteria by the American Diabetes Association: HbA1c >6.5% (48 mmol/mol), fasting glucose 126 mg/dL (≥7 mmol/L) or oral glucose tolerance test measurement 200 mg/dL (≥11 mmol/L).^21^ Baseline CHD, defined variably across studies, included at least myocardial infarction or angina (see Table S1 for details).

### 2.3 Definition of outcomes

The primary outcome was a composite of time to CVD event (nonfatal myocardial infarction, nonfatal stroke or cardiovascular death) or all‐cause mortality. We included all‐cause mortality rather than CVD‐related death alone given that older adults are at high mortality risk from various causes. The time‐to‐event analysis was censored at the time of the earliest occurrence of a CVD event, death, loss to follow‐up or end of follow‐up. Secondary outcomes were the components of the composite, that is, CVD events and all‐cause mortality.

### 2.4 Statistical analysis

We imputed missing exposure and covariate data for each study using multiple imputation by chained equations and produced 20 complete datasets. Coefficients from survival models described below were combined across the imputed datasets using Rubin's rules.^22^

To assess the association between T2D and CVD events and all‐cause mortality, we produced study‐specific cumulative incidence curves stratified by T2D status and performed a two‐stage meta‐analysis to estimate the association. We estimated hazard ratios (HR) and 95% confidence intervals (CIs) by fitting proportional hazards flexible parametric survival models (“Royston‐Parmar” models) in each study.^23^ For the CVD events outcome, a cause‐specific cumulative incidence function competing‐risk model was used with non‐CVD death considered as a competing event.^24^ Study‐specific HRs were combined using a random effects meta‐analysis^25^ and displayed with forest plots. We quantified heterogeneity using I^2^, τ^2^ and via prediction intervals.

For the assessment of CHD risk equivalence, we categorized participants into four groups based on baseline presence of T2D and CHD: (i) no T2D or CHD, (ii) T2D but no CHD, (iii) CHD but no T2D and (iv) both T2D and CHD. We produced study‐specific cumulative incidence curves stratified by participant group and computed HRs for individuals with T2D but no CHD versus CHD but no T2D using the same methods described above.

Descriptions of the additional analyses on the association between HbA1c and outcomes are available in the Appendix S1.

All models were adjusted for established CVD risk factors including age, gender, body mass index, smoking, alcohol consumption, prior CVD, as well as use of antihypertensive and cholesterol‐lowering drugs (including statins). Covariate definitions (Table S2) and details on splines modelling for continuous variables (Appendix S1) are described in the Supplement. For the analyses on CHD risk equivalence, adjustment was made for prior stroke instead of prior CVD.

We conducted prespecified subgroup analyses by age (<75 years vs. ≥75 years), gender (women vs. men) and prior CVD (yes vs. no). Effect modification was assessed by meta‐analysing study‐specific interaction terms of the exposure with the subgroup variables. We performed a series of prespecified and post hoc sensitivity analyses to assess robustness of our findings and explore potential sources of heterogeneity due to diabetes treatment and diabetes duration. Details are available in the Appendix S1.

Analyses were performed using R statistical software version 4.3.2. R packages used to conduct the analyses are listed in the Appendix S1.

## 3 RESULTS

We included individual participant data from a total of 82,723 participants. Study characteristics are summarized in Table 1. Median age across studies was 71 years (range: 65–104) and 55% of participants were women. At baseline, 20% of participants had T2D, 26% had CVD and 17% had CHD. Overall, across all studies, a total of 465,038 person‐years of follow‐up were accumulated. During follow‐up, 29,474 (36%) participants experienced the composite outcome. Additionally, 14,112 (17%) and 24,941 (30%) participants experienced the outcomes of CVD events and all‐cause mortality, respectively. Baseline characteristics by diabetes status and for the analyses on HbA1c are presented in Tables S3 and S4, respectively. The proportion of missing data for each variable and descriptive statistics of baseline characteristics after imputation are summarized in Table S5.

**TABLE 1:** Study characteristics at baseline of adults ≥65 years old.

Characteristics	CoLaus (*N* = 915)	HealthABC (*N* = 3075)	HRS (*N* = 16,781)	OPERAM (*N* = 1980)	SHARE (*N* = 59,972)	Overall (*N* = 82,723)
Age, years	70 [65, 75]	73 [68, 80]	71 [65, 104]	79 [70, 99]	70 [65, 104]	71 [65, 104]
Women	490 (53.6%)	1584 (51.5%)	9687 (57.7%)	883 (44.6%)	32,741 (54.6%)	45,385 (54.9%)
Type 2 diabetes	124 (13.6%)	719 (23.4%)	4573 (27.3%)	634 (32.0%)	10,097 (16.8%)	16,147 (19.5%)
Diabetes duration, years	0 [0, 34]	3 [0, 69]	9 [0, 86]	13 [0, 47]	11 [0, 92]	10 [0, 92]
Current smoker	164 (17.9%)	318 (10.3%)	1778 (10.6%)	157 (7.9%)	5550 (9.3%)	7967 (9.6%)
Weekly alcohol consumption, g ethanol	55 [0, 836]	0 [0, 392]	0 [0, 1760]	0 [0, 770]	8 [0, 5005]	4 [0, 5005]
BMI, kg/m^2^	26.2 [15.5, 51.7]	26.9 [14.6, 52.0]	28.1 [11.0, 78.0]	26.2 [13.2, 98.0]	26.5 [12.5, 98.6]	26.8 [11.0, 98.6]
Antihypertensive treatment	396 (43.3%)	1672 (54.4%)	10,045 (59.9%)	1756 (88.7%)	26,958 (45.0%)	40,827 (49.4%)
Cholesterol‐lowering treatment	242 (26.4%)	437 (14.2%)	7828 (46.6%)	1135 (57.3%)	15,906 (26.5%)	25,548 (30.9%)
Systolic blood pressure, mm Hg	139 [92, 218]	134 [77, 224]	131 [69, 233]	130 [62, 234]	NA	132 [62, 234]
Total cholesterol, mmol/L	5.8 [2.1, 8.9]	5.2 [2.0, 11.4]	4.9 [1.6, 10.7]	3.8 [0.9, 9.9]	NA	5.0 [0.9, 11.4]
HDL cholesterol, mmol/L	1.6 [0.8, 3.7]	1.3 [0.3, 4.2]	1.3 [0.3, 4.9]	1.1 [0.1, 4.4]	NA	1.3 [0.1, 4.9]
Prior CVD	76 (8.3%)	921 (30.0%)	5883 (35.1%)	1267 (64.0%)	13,368 (22.3%)	21,515 (26.0%)
Coronary heart disease	52 (5.7%)	661 (21.5%)	1536 (9.2%)	675 (34.1%)	10,962 (18.3%)	13,886 (16.8%)
Stroke	13 (1.4%)	247 (8.0%)	1633 (9.7%)	518 (26.2%)	3795 (6.3%)	6206 (7.5%)
Follow‐up time, years	13.4 [0.3, 18.0]	13.1 [0.0, 17.4]	6.9 [0.0, 15.2]	1.0 [0.0, 1.7]	4.8 [0.0, 14.6]	5.5 [0.0, 18.0]

*Note*: Values displayed as median [range] or *n* (%).

Abbreviations: BMI, body mass index; CVD, cardiovascular disease; HbA1c, haemoglobin A1c; HDL, high‐density lipoprotein; NA, not available.

### 3.1 Type 2 diabetes and cardiovascular disease events and all‐cause mortality

Cumulative incidence curves for the composite outcome are shown in Figure S3. The incidence rate per 100 person‐years ranged from 4.1 (CoLaus) to 26.0 (OPERAM) in individuals without T2D and from 6.3 (CoLaus) to 33.8 (OPERAM) in individuals with T2D (Table S6).

**FIGURE 1:**
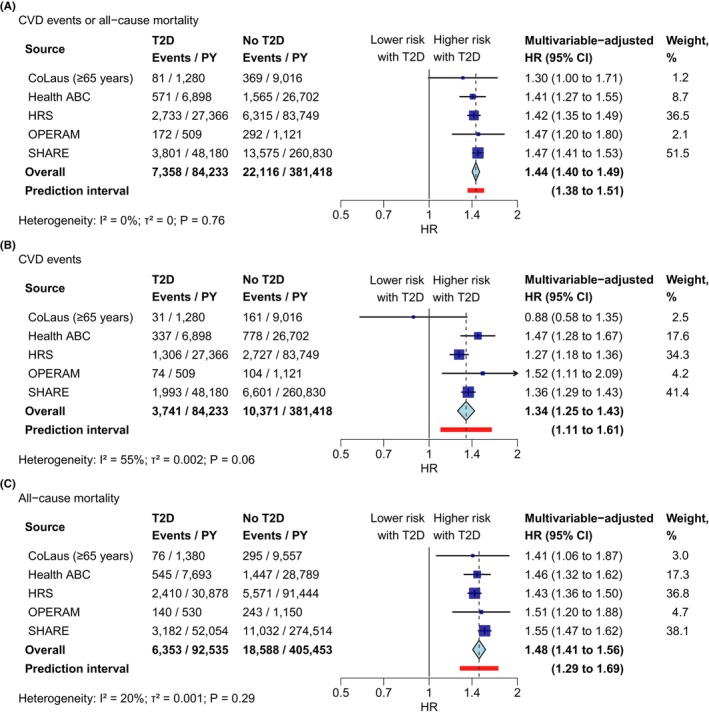
Association between T2D and CVD events and all‐cause mortality outcomes. HR >1 indicates increased risk in individuals with T2D. Study‐specific hazard ratios were estimated using flexible parametric survival models adjusted for age, gender, BMI, smoking, alcohol consumption, prior CVD, use of antihypertensive drugs and use of cholesterol‐lowering drugs. A competing‐risk model was used for the CVD events outcome (Panel B). Overall hazard ratios were calculated using a random‐effects meta‐analysis. BMI, body mass index; CVD, cardiovascular disease; HR, hazard ratio; PY, person‐years at risk; T2D, type 2 diabetes.

We found strong evidence that T2D was associated with increased risk of CVD events or all‐cause mortality (HR 1.44, 95% CI 1.40–1.49; Figure 1A). There was no evidence of heterogeneity across studies. T2D was also associated with increased risk of both components of the composite outcome. The HR was 1.34 (95% CI 1.25–1.43; Figure 1B) for CVD events and 1.48 (95% CI 1.41–1.56; Figure 1C) for all‐cause mortality.

**FIGURE 2:**
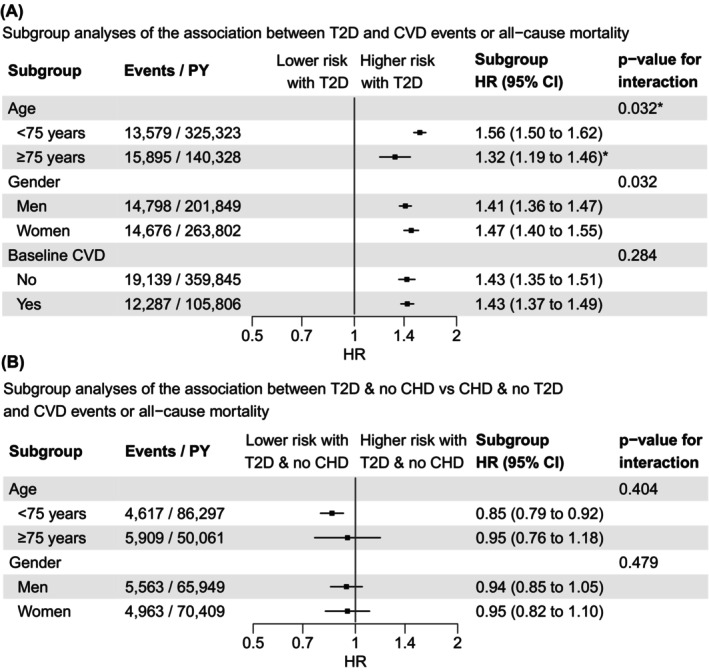
Association between (A) T2D versus no T2D and (B) T2D and no CHD versus CHD and no T2D and CVD events or all‐cause mortality among subgroups. Subgroup hazard ratios were estimated for each study using flexible parametric survival models adjusted for the same covariates as the main model and combined using a random‐effects meta‐analysis. For the interaction *p*‐values, interaction terms between the exposure and the subgroup were included in the study‐specific models and meta‐analysed using a random‐effects model. *Excluding CoLaus as the maximum age was 75 years in this cohort. CHD, coronary heart disease; CVD, cardiovascular disease; HR, hazard ratio; T2D, type 2 diabetes; PY, person‐years at risk.

We found evidence for effect modification by age (<75 years vs. ≥75 years old) on the relative effect scale (*p*‐value for interaction = .032; Figure 2A). Although the risk of CVD events or all‐cause mortality was increased in both age groups, the association was stronger for individuals aged 65–74 years (HR 1.56, 95% CI 1.50–1.62; Figure S4A) compared to those aged ≥75 years at baseline (HR 1.32, 95% CI 1.19–1.46; Figure S4B). We also found evidence for a difference by age for the CVD events outcome (*p*‐value for interaction <.001; Table S7). There was also evidence suggesting an interaction with gender on the composite outcome (HR in men: 1.41, 95% CI 1.36–1.47; HR in women: 1.47, 95% CI 1.40–1.55; *p*‐value for interaction = .032; Figure 2A). No evidence was found for a difference by presence of baseline CVD (*p*‐value for interaction = .284; Figure 2A).

Sensitivity analyses using models adjusted for different sets of covariates (Figures S5 and S6), using a noncompeting‐risk flexible survival parametric model for the CVD events outcome (Figure S7), and excluding the OPERAM study (Table S8) yielded consistent results.

### 3.2 Coronary heart disease risk equivalence

Cumulative incidence curves for the composite outcome for the four groups of individuals (i) without T2D or CHD, (ii) with T2D but no CHD, (iii) with CHD but no T2D and (iv) with both T2D and CHD are presented in Figure S8. The incidence rate per 100 person‐years was lowest in individuals without T2D or CHD (range: 4.0 [CoLaus]–23.5 [OPERAM]) and highest in individuals with both T2D and CHD (range: 9.3 [CoLaus]–42.2 [OPERAM]; Table S6).

**FIGURE 3:**
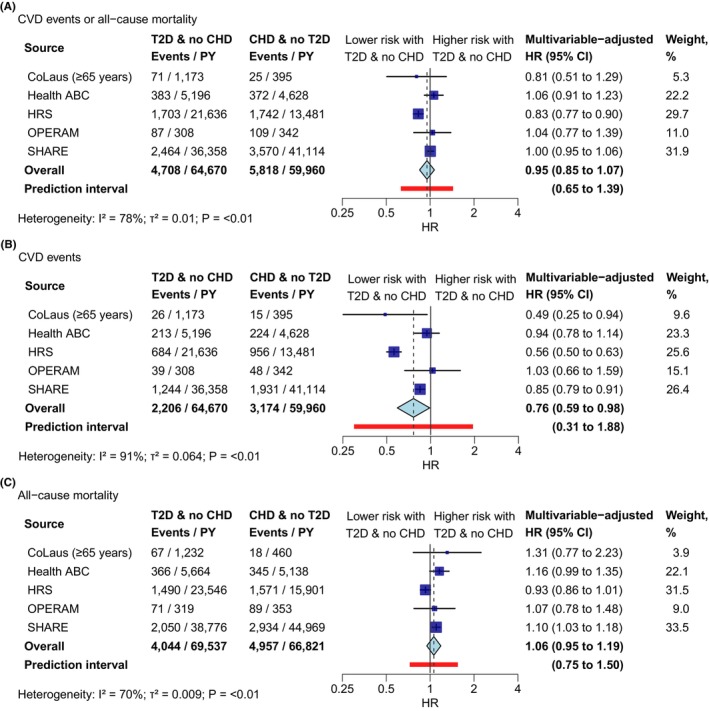
Association between T2D and no CHD versus CHD and no T2D and CVD events and all‐cause mortality outcomes. HR >1 indicates increased risk in individuals with T2D and no CHD. T2D and no CHD: Participants with T2D but no established CHD at baseline; CHD and no T2D: Participants with established CHD but no T2D at baseline. Study‐specific hazard ratios were estimated using flexible parametric survival models adjusted for age, gender, BMI, smoking, alcohol consumption, prior stroke, use of antihypertensive drugs and use of cholesterol‐lowering drugs. A competing‐risk model was used for the CVD events outcome (Panel B). Overall hazard ratios were calculated using a random‐effects meta‐analysis. BMI, body mass index; CHD, coronary heart disease; CVD, cardiovascular disease; HR, hazard ratio; PY, person‐years at risk; T2D, type 2 diabetes.

The HR for the composite outcome of CVD events or all‐cause mortality was 0.95 (95% CI 0.85–1.07) for individuals with T2D but no CHD compared to those with CHD but no T2D (Figure 3A). Heterogeneity was high across the studies (*I* ^2^ = 78%), primarily driven by the two largest cohorts HRS (HR 0.83; 95% CI 0.77–0.90) and SHARE (HR 1.00; 95% CI 0.95–1.06). We found a differential association for CVD events versus all‐cause mortality. Individuals with T2D but no CHD were associated with a lower incidence of CVD events compared to individuals with CHD but no T2D (HR 0.76, 95% CI 0.59–0.98; Figure 3B), while there was almost no evidence for a difference in the risk of all‐cause mortality (HR 1.06, 95% CI 0.95–1.19; Figure 3C).

Subgroup analyses of the composite outcome did not provide evidence for effect modification by age (*p*‐value for interaction = .404; Figure 2B) or gender (*p*‐value for interaction = .479; Figure 2B). There was also no evidence for a difference by age for the CVD events outcome (Table S7). Post hoc subgroup analyses provided weak evidence for a potential stronger association in patients using cholesterol‐lowering drugs compared to those not using cholesterol‐lowering drugs (HR 0.74 [95% CI 0.54–1.01] versus HR 0.81 [95% CI 0.67–0.99]; *p*‐value for interaction = .081). Sensitivity analyses adjusting for different sets of covariates (Figures S9 and S10), or using a noncompeting‐risk flexible survival parametric model for the CVD events outcome (Figure S11), yielded results similar to the main analyses. Excluding the study with the shortest follow‐up, OPERAM, did not change results (Table S8). Post hoc analyses on the CVD events outcome with T2D categorized by diabetes treatment (treated vs. untreated diabetes) or diabetes duration (<5 years, 5–<10 years, ≥10 years) gave similar HRs across all categories with no reduction in heterogeneity between studies (Table S9). Conclusions were also unchanged in post hoc analyses comparing patients with T2D but no history of CVD (including CHD and stroke) to patients with history of CVD but no T2D (Figure S12).

### 3.3 HbA1c and cardiovascular disease events and all‐cause mortality

**FIGURE 4:**
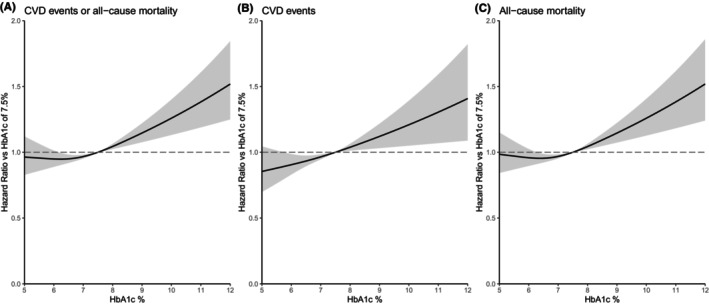
Association between HbA1c levels and CVD events and all‐cause mortality outcomes in older adults with T2D. Hazard ratios for HbA1c were estimated for each study using flexible parametric survival models adjusted for age, gender, BMI, smoking, alcohol consumption, prior CVD, use of antihypertensive drugs and use of cholesterol‐lowering drugs. A competing‐risk model was used for the CVD events outcome (Panel B). HbA1c was modelled as a continuous variable using splines with three knots at the 10th, 50th and 90th percentile. Spline coefficients were combined using a multivariate random‐effects meta‐analysis model. Hazard ratios and confidence intervals were calculated in reference to an HbA1c value of 7.5%. BMI, body mass index; CVD, cardiovascular disease; HbA1c, haemoglobin A1c; T2D, type 2 diabetes.

Figure 4 presents the HR of different HbA1c values compared to a reference of 7.5% in older adults with T2D. There was a nonlinear relationship with the composite outcome; HbA1c values between 6.0% and 7.0% were associated with the lowest risk of CVD events or all‐cause mortality (Figure 4A). Regarding CVD events, analyses revealed an approximately linear relationship with HbA1c (Figure 4B). Analysis using a linear model gave a HR of 1.07 (95% CI 1.02–1.12) for each 1%‐point increase in HbA1c. All‐cause mortality was lowest for HbA1c values between 6.0% and 7.0% (Figure 4C).

Subgroup analyses on the composite outcome did not show evidence for a difference by age, gender or presence of baseline CVD (Figure S13). Further investigation of the CVD events outcome also did not provide evidence for a difference by age (Figure S14). Consistent with the main analysis, analysis using categorized HbA1c demonstrated higher risks for CVD events or all‐cause mortality for HbA1c levels ≥7.5%–<8.4% (HR 1.18; 95% CI 1.05–1.32) and ≥8.5% (HR 1.34; 95% CI 1.17–1.54) compared to levels <7.5%. Sensitivity analyses excluding participants using insulin, sulfonylureas or glinides (Figure S15) and, for the CVD events outcome, using a noncompeting‐risk survival model did not substantially change results (Figure S16).

Results for individuals without T2D and for the overall population are shown in Figures S17 and S18, respectively. The relationship between HbA1c and CVD events and all‐cause mortality was J‐shaped in both populations with HbA1c values of 5.0%–5.7% being associated with the lowest risk of these outcomes.

## 4 DISCUSSION

In this analysis of individual participant data from five prospective studies, we firstly confirmed that T2D was associated with increased risk of CVD events and all‐cause mortality in older adults. However, the magnitude of risk for these outcomes was lower in individuals aged ≥75 years compared to those aged 65–74 years. Secondly, we found that presence of T2D without established CHD conferred a lower risk for CVD events as having established CHD without T2D, suggesting that T2D may not be a CHD risk equivalent in older adults. Thirdly, we found that HbA1c values below 7.5% were associated with lower risk of CVD events and all‐cause mortality compared to values of 7.5% and above among older adults with T2D.

Our finding that older adults with T2D but no CHD have on average a lower risk for future CVD events compared to those with established CHD without T2D challenges some current guidelines that consider T2D as a CHD risk equivalent.^6^, ^7^ Our results contrast those of two studies in older adults that demonstrated a comparable risk between diabetes and established CHD,^9^, ^10^ and are in line with other studies in primarily middle‐aged populations^8^ and older men.^11^ Unlike previous studies in older adults, our study was not limited by exclusion of nonfatal CVD events,^9^ lack of cause‐specific death data^10^ or single‐gender focus.^11^ To our knowledge, our study is the first individual participant data analysis of multiple studies to assess whether T2D is a CHD risk equivalent.

When we assessed the association between T2D but no CHD versus CHD but no T2D and CVD events and all‐cause mortality outcomes, we found high heterogeneity across studies. However, this heterogeneity mainly stemmed from the lack of overlap in confidence intervals among studies due to very precise estimates. Indeed, all study results were consistent in direction for the CVD events outcome, except for the OPERAM study, whose effect, however, was imprecise and had minimal contribution to the overall results (Table S8). Our findings are relevant for CVD risk assessment in older adults with T2D^26^ and strengthen recent 2023 CVD prevention guidelines from the European Society of Cardiology recommending risk stratification using scores to facilitate decisions on treating patients with T2D in primary prevention.^27^ Particularly, patients with T2D but without major other cardiovascular risk factors are not considered equivalent to patients with established CVD.

Consistent with previous research,^2^, ^3^ we found that older adults with T2D had a higher risk of CVD events and mortality compared to older adults without T2D. However, the strength of association for CVD events in the present study (HR 1.34, 95% CI 1.25–1.43) was lower than a previous meta‐analysis (HR of approximately 2 for CHD and stroke outcomes in older age groups),^2^ potentially influenced by our incorporation of more recent data. It is known that the association between presence of T2D and incidence of CVD has decreased over time,^28^ possibly due to multifactorial approaches in CVD prevention guidelines, including management of lipids, blood pressure and glycemia. Unlike previous studies, we used a competing‐risk model, accounting for non‐CVD deaths as competing events, which more accurately estimates the association between T2D and incidence of CVD events.^29^ We also found a weaker association for participants aged ≥75 years compared to 65–74 years at baseline. Notably, previous studies have shown that CVD and mortality risks attenuate with older age at diabetes diagnosis.^30^

Our finding of a nonlinear relationship between HbA1c and all‐cause mortality is consistent with previous studies.^31^, ^32^ Regarding CVD events, our results demonstrate that risk increased continuously with increasing HbA1c values, in agreement with a previous study in older adults.^32^ While our results suggests lower CVD and mortality risk with HbA1c levels <7.5%, our study was not designed to assess causality. Results from clinical trials indicate that intensive glycemic control (HbA1c <6.0 to 6.5%) may increase mortality and the risk for hypoglycemia.^33^ Further research should investigate whether current guideline recommendations for higher HbA1c targets of 8%–9% in older adults with poor or complex health^34^ are safe in terms of CVD events and all‐cause mortality.

Our study has several strengths, including a large sample size of older adults and a long follow‐up which increased statistical power. Access to individual participant data enabled us to standardize variable definitions, model nonlinear associations and explore heterogeneity via subgroup analyses. Our study also has certain limitations. First, we did not conduct a systematic literature review to identify studies to be included in the analysis; this study was conducted with readily available cohorts of older adults for pragmatic reasons. We acknowledge that our included studies are mainly from the United States and Europe and findings need to be confirmed in other settings. Second, our included cohorts might not be representative of current populations in terms of T2D management using SGLT2 inhibitors or GLP1 receptor agonists which have shown to reduce CVD events in clinical trials.^27^ Thus, associations may be weaker in more recent studies. However, the impact of intensive diabetes therapy in older adults is controversial.^26^ Future research should evaluate the cardiovascular preventative effects of these novel antidiabetic medications in older populations. Third, CVD events were self‐reported in the HRS and SHARE cohorts, which may have resulted in misclassification of the outcome. However, this would likely be nondifferential, potentially biasing the estimates towards the null, while we found an increased risk of CVD events. Fourth, due to differences in data collection across studies, we were unable to include further cardiovascular risk factors such as education in our analyses. Future studies should also evaluate additional interactions of interest, for example, with chronic kidney disease, to further explore CHD risk equivalence in patients with diabetes.

In conclusion, in this large individual participant data analysis of >80,000 older adults, we found that T2D was associated with increased risk of CVD events and all‐cause mortality in older adults aged ≥65 years, but the magnitude of risk was smaller in individuals aged ≥75 years compared to those aged 65–74 years. Individuals with T2D without CHD had lower risk of CVD events compared to individuals with CHD without T2D. Our results suggest that T2D may not be a CHD risk equivalent for CVD events in older adults.

## AUTHOR CONTRIBUTIONS

Del Giovane, Rodondi, Waeber, Gencer, Aponte Ribero and Efthimiou were involved in concept and design. All authors were involved in acquisition, analysis or interpretation of data. Aponte Ribero, Gencer, Del Giovane and Efthimiou were involved in drafting of the manuscript. All authors were involved in critical revision of the manuscript for important intellectual content. Aponte Ribero was involved in statistical analysis. Del Giovane, Waeber and Gencer obtained funding. Abolhassani was involved in administrative, technical or material support. Gencer, Del Giovane and Efthimiou were involved in supervision.

## CONFLICT OF INTEREST STATEMENT

OE received consulting fees from Biogen Pharmaceuticals, paid to the University of Bern, irrelevant to this project. AC received honoraria payments for a lecture from Novo Nordisk, irrelevant to this project. The authors declare that there are no other relationships or activities that could appear to have influenced the submitted work.

## Supporting information


Appendix S1.
