# Supplementary material for: Diabetes and the risk of cardiovascular events and all‐cause mortality among older adults: an individual participant data analysis of five prospective studies
Source: Eur J Clin Invest. 2024 Oct 28;55(2):e14340. doi: 10.1111/eci.14340 (PMC11744924; doi:10.1111/eci.14340)
Supplement: Supplementary file 1 — Appendix S1. [file ECI-55-e14340-s001.docx]

**Supplemental Material**

Supplement to: Diabetes and the risk of cardiovascular events and all-cause mortality among older adults: an individual participant data analysis of five prospective studies

**Contents**

[Supplemental Methods 3](#_Toc175832174)

[Description of included studies 3](#_Toc175832175)

[Power estimation 4](#_Toc175832176)

[Analyses to assess the association between HbA1c and cardiovascular disease (CVD) events and all-cause mortality 4](#_Toc175832177)

[Modelling of continuous covariates 4](#_Toc175832178)

[Sensitivity analyses 5](#_Toc175832179)

[Software 5](#_Toc175832180)

[Table S1 Definition of exposures by study 6](#_Toc175832181)

[Table S2 Definition of covariates 7](#_Toc175832182)

[Table S3 Study characteristics by type 2 diabetes status 8](#_Toc175832183)

[Table S4 Study characteristics at baseline for the analyses on the association between HbA1c levels and outcomes 9](#_Toc175832184)

[Table S5 Proportion of missing data and summary statistics of patient characteristics after multiple imputation 10](#_Toc175832185)

[Table S6 Incidence rate of outcomes by T2D and CHD status 11](#_Toc175832186)

[Table S7 Age subgroup analyses for CVD events outcome 11](#_Toc175832187)

[Table S8 Results excluding OPERAM 12](#_Toc175832188)

[Table S9 Sensitivity analysis on the association between T2D & no CHD vs CHD & no T2D and CVD events; T2D categorized by diabetes treatment (untreated vs treated) and diabetes duration (<5 years vs 5-<10 years vs ≥10 years) 13](#_Toc175832189)

[Figure S1 Participant flow diagram 14](#_Toc175832190)

[Figure S2 Participant flow diagram (analyses on HbA1c) 15](#_Toc175832191)

[Figure S3 Cumulative incidence curves by study and T2D status for composite outcome of CVD events or all-cause mortality 16](#_Toc175832192)

[Figure S4 Association between T2D and primary outcome of CVD events or all-cause mortality by study in individuals A) <75 years and B) ≥75 years of age 17](#_Toc175832193)

[Figure S5 Association between T2D and outcomes using an age- and gender-adjusted model 18](#_Toc175832194)

[Figure S6 Association between T2D and outcomes using a fully adjusted model* 19](#_Toc175832195)

[Figure S7 Association between T2D and secondary outcome of CVD events using a non-competing-risk flexible parametric survival model 20](#_Toc175832196)

[Figure S8 Cumulative incidence curves by study and T2D and CHD status for composite outcome of CVD events or all-cause mortality 21](#_Toc175832197)

[Figure S9 Association between T2D & no CHD vs CHD & no T2D and outcomes using an age- and gender-adjusted model 22](#_Toc175832198)

[Figure S10 Association between T2D & no CHD vs CHD & no T2D and outcomes using a fully adjusted model* 23](#_Toc175832199)

[Figure S11 Association between T2D & no CHD vs CHD & no T2D and CVD events using a non-competing-risk flexible parametric survival model 24](#_Toc175832200)

[Figure S12 Association between T2D & no CVD* vs CVD* & no T2D and outcomes 25](#_Toc175832201)

[Figure S13 Subgroup analyses of the association between HbA1c and the composite outcome of CVD events or all-cause mortality in the T2D subpopulation 26](#_Toc175832202)

[Figure S14 Age subgroup analyses of the association between HbA1c and the CVD events outcome in the T2D subpopulation 27](#_Toc175832203)

[Figure S15 Association between continuous HbA1c and CVD events or all-cause mortality in the T2D subpopulation; excluding patients using insulin, sulfonylureas or glinides 28](#_Toc175832204)

[Figure S16 Association between HbA1c and CVD events using a non-competing-risk flexible parametric survival model 29](#_Toc175832205)

[Figure S17 Association between HbA1c and outcomes in older adults without T2D 30](#_Toc175832206)

[Figure S18 Association between HbA1c and outcomes in older adults with or without T2D 31](#_Toc175832207)

[References 32](#_Toc175832208)

# Supplemental Methods

This study was reported in accordance with the STrengthening the Reporting of OBservational studies in Epidemiology (STROBE) statement.^1^

## Description of included studies

*Cohorte Lausannoise (CoLaus)*

CoLaus is a population-based cohort study of 6,188 individuals aged 35 to 75 years living in Lausanne, Switzerland. The primary objective of the study, initiated in 2003, was to investigate the epidemiology and genetic determinants of cardiovascular risk factors and metabolic syndrome.^2^ Hemoglobin A1c (HbA1c) data was first collected during the second follow-up period, which occurred between 2014 and 2018. For our current study, we included 916 patients aged ≥65 years at baseline. For the analyses on HbA1c, we considered the second follow-up (2014 to 2018) as the baseline (time zero), corresponding to 1,540 participants aged ≥65 years at second follow-up.

Information on how researchers can send requests to access the dataset are available on the study website: <https://www.colaus-psycolaus.ch/professionals/how-to-collaborate/>

*Health, Aging, and Body Composition (Health ABC)*

Health ABC is a prospective longitudinal study of 3,075 individuals aged 70 to 79 years recruited in 1997-98 in the USA, investigating risk factors for the decline of function and body composition with age in healthier older persons.^3^ We included all 3,075 individuals for our study. Regarding the analyses on HbA1c, the coordinating center advised against the use of HbA1c data from Year 1 (baseline) of the study for longitudinal analyses due to potential measurement errors. Consequently, we used Year 6 as the baseline (time zero), resulting in the inclusion of data from 2,617 participants for the analyses on HbA1c.

Information on how researchers can send requests to access the dataset are available on the study website: <https://healthabc.nia.nih.gov/>

*Health and Retirement Study (HRS)*

HRS is a nationally representative longitudinal panel study of over 37,000 individuals aged 50 and older in the USA.^4^ The study was set up to provide data on the changing health and economic circumstances associated with ageing. Surveys are conducted every two years, with the addition of new cohorts every six years. Since 2006, data collection included biomarkers such as HbA1c and cholesterol, and questions on the use of cholesterol-lowering medication. For the current study, we defined baseline (time zero) as the first survey starting from 2006 onwards when biomarker data was first collected, at which the participant was aged 65 years or older.^5-26^ We included data from 16,824 participants for this study.

Data from this study are available free of charge for researchers after registration on: <https://hrs.isr.umich.edu/>

*Optimising Therapy to Prevent Avoidable Hospital Admissions in Multimorbid Older People (OPERAM)*

OPERAM is a multicentre randomized controlled trial conducted to assess the effectiveness of a computer software, the Systematic Tool to Reduce Inappropriate Prescribing assistant, in reducing drug-related hospital admissions.^27^ The trial included 2,008 participants aged 70 years or older with multimorbidity (three or more chronic conditions) and polypharmacy (five or more regular medications) and was conducted in four European countries (Switzerland, Netherlands, Belgium, Republic of Ireland) We included data from both randomization arms, assuming no impact of intervention on our study outcomes based on the negative results on drug-related hospital admissions and all-cause mortality during the trial period.^27^ Baseline was defined as the time of enrolment. Data from 638 patients with type 2 diabetes (T2D) contributed to the analysis on HbA1c, as HbA1c measurements were only collected in diabetic participants.

Requests from researchers to access the dataset may be sent to [operam@biham.unibe.ch](mailto:operam@biham.unibe.ch).

*Survey of Health, Ageing and Retirement in Europe (SHARE)*

SHARE is a longitudinal panel study on over 140,000 individuals aged 50 or older in 27 European countries and Israel, providing longitudinal data on economic, social, and health factors and the ageing processes.^28,29^ Surveys have been conducted every two years since 2004. Information on the date of past CVD events was first collected in the fourth survey period (wave 4).^30-35^ Consequently, for our current study we included data from the second survey period (wave 2, conducted in 2006) onwards,^36-41^ excluding wave 3 as no relevant information for our study was collected during this round. We defined baseline (time zero) as the first survey, starting from wave 2 (year 2006), when the participant was aged 65 years or older. A total of 59,972 participants contributed to our study except for the analyses on HbA1c where data were not available.

Data from this study are available free of charge for researchers after registration on: <https://share-eric.eu/data/>

## Power estimation

Power calculations to detect an increased risk of the composite outcome (CVD event or all-cause mortality) were conducted prior to commencing analyses.^42^ Assuming a 5-year risk between 12% to 25% with a minimum relative risk of 1.2 for older adults with T2D versus without T2D, power was at least 99% (alpha = 0.05; two-sided test).

## Analyses to assess the association between HbA1c and cardiovascular disease (CVD) events and all-cause mortality

We used a two-stage meta-analysis to assess the association between HbA1c and CVD event and all-cause mortality outcomes. We modelled HbA1c as a continuous variable using restricted cubic splines with 3 knots at the 10^th^, 50^th^ and 90^th^ percentiles in the T2D and no T2D subpopulations and 4 knots at the 5^th^, 35^th^, 65^th^ and 95^th^ percentiles in the overall population; selection of the number of knots was based on the model for the primary outcome using Akaike information criterion.

We used flexible parametric survival models for the primary outcome and all-cause mortality, and a cause-specific cumulative incidence function competing-risk model for CVD events with non-CVD death as a competing event.^43^ Study‐specific estimates were combined in a multivariate random effects meta-analysis.^44^ Analyses were stratified by the presence of T2D at baseline. Hazard ratios of HbA1c values were displayed versus a reference value of 7.5% for the subpopulation of individuals with T2D (common HbA1c goal for older adults^45^) and 5.7% for the overall population and the subpopulation of individuals without T2D (cut-off for normoglycemia^46^).

## Modelling of continuous covariates

We modelled continuous covariates body mass index, systolic blood pressure (SBP), alcohol consumption, total cholesterol and high-density lipoprotein (HDL) cholesterol using restricted cubic splines with 3 knots at the 10^th^, 50^th^ and 90^th^ percentiles. For alcohol consumption we used the 10^th^, 75^th^ and 90^th^ percentiles because the 50^th^ percentile was the same value as the 10^th^ percentile, except for SHARE where a linear term was used due to model convergence issues. A linear term was included for age as graphical checks did not indicate a nonlinear association with the primary outcome.

## Sensitivity analyses

The following pre-specified sensitivity analyses were conducted: we ran models for the primary outcome (i) adjusting only for age and gender, and (ii) additionally adjusting for SBP, total cholesterol and HDL cholesterol, except for the SHARE cohort where those data were not available; for the analyses on the association between HbA1c and CVD events or all-cause mortality, in the subpopulation of individuals with T2D, we (i) categorized baseline HbA1c by <7.5%, ≥7.5% to <8.5% and ≥8.5%,^47^ and (ii) excluded participants using medication conferring a high risk of hypoglycemic events (insulin, sulfonylureas or glinides). The latter analyses could not be conducted using data from HRS as details on diabetic drug types were not available.

In addition, we conducted the following post-hoc sensitivity analyses: we used non-competing-risk (cause-specific hazard) flexible parametric survival models for the secondary outcome of CVD events; we conducted analyses excluding OPERAM as the study with the shortest follow-up; for the analyses of CHD risk equivalence on the CVD events outcome, we further categorized participants with baseline T2D by (i) use of antidiabetic medication (yes vs no) and (ii) duration of diabetes (<5 years, 5-10 years and ≥10 years), in order to explore heterogeneity across studies based on previously identified predictors of CHD risk equivalence;^48^ we analysed CHD risk equivalence by comparing patients with T2D but no CVD (defined as history of CHD or stroke) to patients with CVD but no T2D; for the analyses on the association between HbA1c and CVD events, in the subpopulation of individuals with T2D, we fitted a model where the effect of HbA1c was assumed to be linear.

## Software

Analyses were performed using R statistical software version 4.3.2.^49^ with the mice,^50^ flexsurv,^51^ meta,^52^ and mvmeta^53^ packages.

# Table S1 Definition of exposures by study

|  | **CoLaus** | **Health ABC** | **HRS** | **OPERAM** | **SHARE** |
| --- | --- | --- | --- | --- | --- |
| **Type 2 diabetes** | Self-reported diabetes diagnosis, diabetes medication use, or fasting plasma glucose ≥7.0 mmol/L. | Self-reported diabetes diagnosis, diabetes medication use, fasting glucose ≥126 mg/dL, or oral glucose tolerance test ≥11 mmol/l. | Self-reported diabetes diagnosis, diabetes medication use, HbA1c ≥6.5%, or fasting glucose ≥126 mg/dL. | Type 2 diabetes diagnosis based on medical records (ICD-10 code E11), or diabetes medication use (ATC code A10). | Self-reported diabetes diagnosis, or diabetes medication use. |
| **Coronary heart disease** | Prevalent coronary artery disease, angina, or myocardial infarction. | Self-report diagnosis or inpatient and outpatient HCFA data of bypass/CABG, angioplasty, myocardial infarction, or angina. | Self-reported diagnosis of heart attack/ myocardial infarction or angina/ chest pains. | Diagnosis of ischemic heart diseases based on medical records (ICD-10 codes I20-25). | Self-reported diagnosis of heart attack including myocardial infarction or coronary thrombosis or any other heart problem including congestive heart failure. |
| **HbA1c** | Measured from whole blood by high performance liquid chromatography using a Bio-Rad, D-10TM system. | Measured from whole blood using the Bio-Rad Variant analyzer. | Measured from dried blood spots using various systems, depending on the year of data collection. NHANES equivalent values were used for the analyses. | Collected from medical records up to 1-year prior to enrolment. | Not available for analysis. |

ATC, Anatomical Therapeutic Chemical; CABG, coronary artery bypass graft; HbA1c, hemoglobin A1C; HCFA, Health Care Financing Administration; ICD-10, International Classification of Diseases 10th Revision

# Table S2 Definition of covariates

| **Covariate** | **Definition** |
| --- | --- |
| BMI | Calculated as the ratio of weight in kg divided to the square of height in meters |
| Prevalent CVD | Presence of heart disease, stroke or peripheral artery disease; self-reported or based on medical records (ICD-10 codes I09.8, I11.0, I13.0, I13.2, I20-25, I50, I60-63, I65-70, I73, I74, I97.0, I97.1, G45-46) |
| Antihypertensive medication | Self-reported use or extracted from medical records (ATC codes C02, C03, C07, C08, C09) |
| Cholesterol-lowering medication | Self-reported use or extracted from medical records (ATC code C10) |
| Smoking | Categorized as current smoker vs no current smoker |
| Alcohol consumption | Alcohol consumption was harmonized across the studies as a continuous variable representing weekly ethanol consumption in grams (g). This was calculated by multiplying the average number of alcoholic drinks per week by the average alcohol content for a standard drink in each respective country. We assumed an average ethanol content per standard drink of 11 g for CoLaus, OPERAM and SHARE (standard drink defined as 10-12 g of ethanol in most European countries), and 14 g for Health ABC and HRS (standard drink defined as 14 g of ethanol in the US)^54^ |

ATC, Anatomical Therapeutic Chemical; ICD-10, International Classification of Diseases 10th Revision

# Table S3 Study characteristics by type 2 diabetes status

| **Characteristics** | **No Type 2 Diabetes**  **(N=66,576)** | **Type 2 Diabetes**  **(N=16,147)** | **Overall**  **(N=82,723)** |
| --- | --- | --- | --- |
|  | Median [range] or n (%) | Median [range] or n (%) | Median [range] or n (%) |
| Age, years | 71 [65, 104] | 71 [65, 102] | 71 [65, 104] |
| Female gender | 37,027 (55.6%) | 8,358 (51.8%) | 45,385 (54.9%) |
| Current smoker | 6,561 (9.9%) | 1,406 (8.7%) | 7,967 (9.6%) |
| Weekly alcohol consumption, g ethanol | 8 [0, 5010] | 0 [0, 5010] | 4 [0, 5010] |
| BMI, kg/m2 | 26.3 [11.0, 98.4] | 28.8 [14.3, 98.6] | 26.8 [11.0, 98.6] |
| Antihypertensive treatment | 29,880 (44.9%) | 10,947 (67.8%) | 40,827 (49.4%) |
| Cholesterol-lowering treatment | 17,524 (26.3%) | 8,024 (49.7%) | 25,548 (30.9%) |
| Systolic blood pressure, mm Hg | 131 [62, 234] | 133 [62, 224] | 132 [62, 234] |
| Total cholesterol, mmol/L | 5.1 [1.4, 11.4] | 4.7 [0.9, 10.7] | 5.0 [0.9, 11.4] |
| HDL cholesterol, mmol/L | 1.4 [0.3, 4.8] | 1.2 [0.1, 4.9] | 1.3 [0.1, 4.9] |
| HbA1c, % | 5.6 [3.0, 8.3]* | 6.7 [4.4, 20.0] | 5.8 [3.0, 20.0] |
| Prior CVD | 15,554 (23.4%) | 5,961 (36.9%) | 21,515 (26.0%) |
| Coronary heart disease | 10,123 (15.2%) | 3,763 (23.3%) | 13,886 (16.8%) |
| Stroke | 4,394 (6.6%) | 1,812 (11.2%) | 6,206 (7.5%) |

*5 patients had an HbA1c value >6.5% but were diagnosed with diabetes other than type 2 diabetes (ICD-10 codes E13 and E14).

BMI, body mass index; CVD, cardiovascular disease; HbA1c, Hemoglobin A1c; HDL, high-density lipoprotein; ICD-10, International Classification of Diseases 10th Revision

# Table S4 Study characteristics at baseline for the analyses on the association between HbA1c levels and outcomes

| **Characteristics** | **CoLaus**  **(N=1,540)** | **Health ABC**  **(N=2,617)** | **HRS**  **(N=16,781)** | **OPERAM***  **(N=634)** | **Overall**  **(N=21,576)** |
| --- | --- | --- | --- | --- | --- |
|  | Median [range] or n (%) | Median [range] or n (%) | Median [range] or n (%) | Median [range] or n (%) | Median [range] or n (%) |
| Age, years | 73 [65, 87] | 78 [73, 85] | 71 [65, 104] | 78 [70, 97] | 73 [65, 104] |
| Female gender | 906 (58.8%) | 1,406 (53.7%) | 9,687 (57.7%) | 253 (39.9%) | 12,254 (56.8%) |
| Type 2 diabetes | 323 (21.0%) | 719 (27.5%) | 4,573 (27.3%) | 634 (100%) | 6,253 (29.0%) |
| HbA1c, % | 5.7 [3.8, 11.2] | 5.6 [4.0, 11.9] | 5.7 [3.0, 20.0] | 7.0 [4.6, 12.4] | 5.7 [3.0, 20.0] |
| Current smoker | 194 (12.6%) | 136 (5.2%) | 1,778 (10.6%) | 45 (7.1%) | 2,153 (10.0%) |
| Weekly alcohol consumption, g ethanol | 33 [0, 847] | 0 [0, 392] | 0 [0, 1760] | 0 [0, 462] | 0 [0, 1760] |
| BMI, kg/m2 | 26.2 [13.9, 46.0] | 26.7 [14.3, 53.7] | 28.1 [11.0, 78.0] | 28.1 [15.4, 98.0] | 27.7 [11.0, 98.0] |
| Antihypertensive treatment | 789 (51.2%) | 1,742 (66.6%) | 10,045 (59.9%) | 595 (93.8%) | 13,174 (61.1%) |
| Cholesterol-lowering treatment | 569 (36.9%) | 735 (28.1%) | 7,828 (46.6%) | 434 (68.5%) | 9,569 (44.4%) |
| Systolic blood pressure, mm Hg | 131 [87, 207] | 136 [86, 230] | 131 [69, 233] | 131 [62, 214] | 132 [62, 233] |
| Total cholesterol, mmol/L | 5.3 [1.9, 9.0] | 4.8 [1.7, 9.2] | 4.9 [1.6, 10.7] | 3.6 [0.9, 7.5] | 4.9 [0.9, 10.7] |
| HDL cholesterol, mmol/L | 1.6 [0.5, 3.7] | 1.4 [0.6, 4.1] | 1.3 [0.3, 4.9] | 1.0 [0.1, 2.3] | 1.4 [0.11, 4.9] |
| Prior CVD | 294 (19.1%) | 956 (36.5%) | 5,883 (35.1%) | 440 (69.4%) | 7,575 (35.1%) |
| Coronary heart disease | 160 (10.4%) | 701 (26.8%) | 1,536 (9.2%) | 257 (40.5%) | 2,654 (12.3%) |
| Stroke | 91 (5.9%) | 301 (11.5%) | 1,633 (9.7%) | 154 (24.3%) | 2,180 (10.1%) |
| Follow-up time, years | 3.8 [0.1, 7.0] | 9.5 [0, 12.3] | 6.9 [0.0, 15.2] | 1.0 [0.0, 1.4] | 6.3 [0, 15.2] |

* Only patients with type 2 diabetes were included for these analyses, since HbA1c data was not collected in patients without diabetes in OPERAM.

BMI, body mass index; CVD, cardiovascular disease; HbA1c, hemoglobin A1c; HDL, high-density lipoprotein

# Table S5 Proportion of missing data and summary statistics of patient characteristics after multiple imputation

| **Characteristics** | **CoLaus** | | **Health ABC** | | **HRS** | | **OPERAM** | | **SHARE** | |
| --- | --- | --- | --- | --- | --- | --- | --- | --- | --- | --- |
|  | n (%) missing | After imputation: median [range] or % | n (%) missing | After imputation: median [range] or % | n (%) missing | After imputation: median [range] or % | n (%) missing | After imputation: median [range] or % | n (%) missing | After imputation: median [range] or % |
| Age, years | 0 (0%) | 70 [65, 75] | 0 (0%) | 73 [68, 80] | 0 (0%) | 71 [65, 104] | 0 (0%) | 79 [70, 99] | 0 (0%) | 70 [65, 104] |
| Female gender | 0 (0%) | 53.6% | 0 (0%) | 51.5% | 0 (0%) | 57.7% | 0 (0%) | 44.6% | 0 (0%) | 54.6% |
| Type 2 diabetes | 0 (0%) | 13.6% | 0 (0%) | 23.4% | 0 (0%) | 27.3% | 0 (0%) | 32.0% | 0 (0%) | 16.8% |
| Current smoker | 0 (0%) | 17.9% | 5 (0.2%) | 10.4% | 7,133 (42.5%) | 17.6% | 8 (0.4%) | 8.0% | 19,716 (32.9%) | 14.7% |
| Weekly alcohol consumption, g ethanol | 0 (0%) | 55 [0, 836] | 152 (4.9%) | 0 [0, 392] | 2,806 (16.7%) | 0 [0, 1764] | 15 (0.8%) | 0 [0, 770] | 12,029 (20.1%) | 8 [0, 5005] |
| BMI, kg/m2 | 0 (0%) | 26.3 [15.5, 51.7] | 0 (0%) | 26.9 [14.6, 52.0] | 1,391 (8.3%) | 28.1 [11.0, 78.0] | 159 (8.0%) | 26.2 [13.2, 98.0] | 2,469 (4.1%) | 26.5 [12.5, 98.6] |
| Antihypertensive treatment | 0 (0%) | 43.3% | 10 (0.3%) | 54.5% | 157 (0.9%) | 60.5% | 0 (0%) | 88.7% | 31 (0.1%) | 45.0% |
| Cholesterol-lowering treatment | 0 (0%) | 26.4% | 10 (0.3%) | 14.2% | 2,126 (12.7%) | 51.1% | 0 (0%) | 57.3% | 139 (0.2%) | 26.6% |
| Systolic blood pressure, mm Hg | 2 (0.2%) | 139 [92, 218] | 0 (0%) | 134 [77, 224] | 4,459 (26.6%) | 131 [69, 233] | 65 (3.3%) | 130 [62, 234] | 59,972 (100%) | NA |
| Total cholesterol, mmol/L | 3 (0.3%) | 5.8 [2.1, 8.9] | 35 (1.1%) | 5.2 [2.0, 11.4] | 4,942 (29.5%) | 4.9 [1.6, 10.7] | 1,245 (62.9%) | 3.7 [0.9, 9.9] | 59,972 (100%) | NA |
| HDL cholesterol, mmol/L | 3 (0.3%) | 1.6 [0.8, 3.7] | 37 (1.2%) | 1.3 [0.3, 4.2] | 6,050 (36.1%) | 1.3 [0.3, 4.9] | 1,264 (63.8%) | 1.1 [0.1, 4.4] | 59,972 (100%) | NA |
| Prior CVD | 0 (0%) | 8.3% | 66 (2.1%) | 30.6% | 0 (0%) | 35.1% | 0 (0%) | 64.0% | 0 (0%) | 22.3% |
| Coronary heart disease | 0 (0%) | 5.7% | 54 (1.8%) | 21.9% | 3,021 (18.0%) | 21.5% | 0 (0%) | 34.1% | 0 (0%) | 18.3% |
| Stroke | 0 (0%) | 1.4% | 0 (0%) | 8.1% | 0 (0%) | 9.7% | 0 (0%) | 26.2% | 0 (0%) | 6.3% |

BMI, body mass index; CVD, cardiovascular disease; HDL, high-density lipoprotein; NA, not available

# Table S6 Incidence rate of outcomes by T2D and CHD status

|  | **Incidence rate per 100 person-years** | | | | | |
| --- | --- | --- | --- | --- | --- | --- |
|  | **T2D** | **No T2D** | **No CHD or T2D** | **T2D but no CHD** | **CHD but no T2D** | **Both T2D and CHD** |
| **CVD event or all-cause mortality** | | | | | | |
| CoLaus | 6.3 | 4.1 | 4.0 | 6.1 | 6.3 | 9.3 |
| Health ABC | 8.3 | 5.9 | 5.4 | 7.4 | 8.0 | 11.0 |
| HRS | 10.0 | 7.5 | 6.5 | 7.9 | 12.9 | 18.0 |
| OPERAM | 33.8 | 26.0 | 23.5 | 28.3 | 31.9 | 42.2 |
| SHARE | 7.9 | 5.2 | 4.6 | 6.8 | 8.7 | 11.3 |
| **CVD event** | | | | | | |
| CoLaus | 2.4 | 1.8 | 1.7 | 2.2 | 3.8 | 4.7 |
| Health ABC | 4.9 | 2.9 | 2.5 | 4.1 | 4.8 | 7.3 |
| HRS | 4.8 | 3.3 | 2.5 | 3.2 | 7.1 | 10.9 |
| OPERAM | 14.5 | 9.3 | 7.2 | 12.7 | 14.0 | 17.4 |
| SHARE | 4.1 | 2.5 | 2.1 | 3.4 | 4.7 | 6.3 |
| **All-cause mortality** | | | | | | |
| CoLaus | 0.5 | 0.3 | 0.3 | 0.5 | 0.3 | 0.5 |
| Health ABC | 0.6 | 0.4 | 0.4 | 0.5 | 0.6 | 0.7 |
| HRS | 0.7 | 0.5 | 0.4 | 0.5 | 0.8 | 1.0 |
| OPERAM | 2.2 | 1.8 | 1.6 | 1.9 | 2.1 | 2.7 |
| SHARE | 0.5 | 0.3 | 0.3 | 0.4 | 0.5 | 0.7 |

CHD, coronary heart disease; CVD, cardiovascular disease; T2D, type 2 diabetes

# Table S7 Age subgroup analyses for CVD events outcome

| **Subgroup** | **Hazard ratio (95% CI)** | **Interaction p-value** |
| --- | --- | --- |
| **Association between T2D and CVD events** | | |
| Age 65-74 years old | 1.45 (1.34 to 1.56) | <0.001* |
| Age ≥75 years old | 1.21 (1.10 to 1.33)* |  |
| **Association between T2D & no CHD vs CHD & no T2D and CVD events** | | |
| Age 65-74 years old | 0.69 (0.52 to 0.91) | 0.206* |
| Age ≥75 years old | 0.79 (0.57 to 1.09)* |  |

Subgroup hazard ratios were estimated for each study using flexible parametric survival models adjusted for the same covariates as the main model and combined using a random-effects meta-analysis. For the interaction p-values, interaction terms between the exposure and the subgroup were included in the study-specific models and meta-analysed using a random-effects model.

* Excluding CoLaus as the maximum age was 75 years in this cohort

CHD, coronary heart disease; CVD, cardiovascular disease; HR, hazard ratio; T2D, type 2 diabetes; PY, person-years at risk

# Table S8 Results excluding OPERAM

| **Outcomes** | **Multivariable-adjusted hazard ratio (95% CI)** |
| --- | --- |
| **Association between T2D and outcomes excluding OPERAM** | |
| CVD event or all-cause mortality | 1.44 (1.40 to 1.49) |
| CVD event | 1.33 (1.24 to 1.43) |
| All-cause mortality | 1.48 (1.40 to 1.56) |
| **Association between T2D & no CHD vs CHD & no T2D and outcomes excluding OPERAM** | |
| CVD event or all-cause mortality | 0.94 (0.82 to 1.08) |
| CVD event | 0.72 (0.54 to 0.96) |
| All-cause mortality | 1.06 (0.94 to 1.20) |

# Table S9 Sensitivity analysis on the association between T2D & no CHD vs CHD & no T2D and CVD events; T2D categorized by diabetes treatment (untreated vs treated) and diabetes duration (<5 years vs 5-<10 years vs ≥10 years)

|  | **N T2D & no CHD** | **N CHD & no T2D** | **Multivariable-adjusted hazard ratio (95% CI)** | **I^2^** | **τ²** | **p-value for Q-test** |
| --- | --- | --- | --- | --- | --- | --- |
| **Untreated diabetes (overall)** | 2,062 | 11,569 | 0.77 (0.55 to 1.07) | 81% | 0.096 | <0.001 |
| CoLaus | 33 | 39 | 0.51 (0.20 to 1.29) | - | - | - |
| Health ABC | 241 | 462 | 0.95 (0.75 to 1.21) | - | - | - |
| HRS | 559 | 2,343 | 0.50 (0.39 to 0.63) | - | - | - |
| OPERAM | 66 | 418 | 1.33 (0.64 to 2.75) | - | - | - |
| SHARE | 1,163 | 8,307 | 0.84 (0.73 to 0.98) | - | - | - |
| **Treated diabetes (overall)** | 9,674 | 11,569 | 0.75 (0.59 to 0.96) | 89% | 0.052 | <0.001 |
| CoLaus | 78 | 39 | 0.48 (0.24 to 0.97) | - | - | - |
| Health ABC | 266 | 462 | 0.93 (0.74 to 1.18) | - | - | - |
| HRS | 2,740 | 2,343 | 0.57 (0.51 to 0.64) | - | - | - |
| OPERAM | 311 | 418 | 0.95 (0.60 to 1.53) | - | - | - |
| SHARE | 6,279 | 8,307 | 0.85 (0.78 to 0.91) | - | - | - |
| **Diabetes duration <5 years (overall)** | 3,036 | 11,569 | 0.75 (0.51 to 1.12) | 88% | 0.159 | <0.001 |
| CoLaus | 53 | 39 | 0.47 (0.20 to 1.09) | - | - | - |
| Health ABC | 277 | 462 | 1.00 (0.79 to 1.26) | - | - | - |
| HRS | 1,103 | 2,343 | 0.48 (0.40 to 0.57) | - | - | - |
| OPERAM | 96 | 418 | 1.58 (0.80 to 3.11) | - | - | - |
| SHARE | 1,464 | 8,307 | 0.72 (0.62 to 0.85) | - | - | - |
| **Diabetes duration 5-<10 years (overall)** | 2,532 | 11,569 | 0.74 (0.53 to 1.05) | 71% | 0.087 | 0.007 |
| CoLaus | 22 | 39 | 0.44 (0.10 to 1.84) | - | - | - |
| Health ABC | 51 | 462 | 1.11 (0.72 to 1.72) | - | - | - |
| HRS | 727 | 2,343 | 0.53 (0.43 to 0.64) | - | - | - |
| OPERAM | 48 | 418 | 1.27 (0.47 to 3.43) | - | - | - |
| SHARE | 1,684 | 8,307 | 0.74 (0.64 to 0.85) | - | - | - |
| **Diabetes duration ≥10 years (overall)** | 6,211 | 11,569 | 0.76 (0.62 to 0.94) | 83% | 0.033 | <0.001 |
| CoLaus | 36 | 39 | 0.52 (0.21 to 1.3) | - | - | - |
| Health ABC | 179 | 462 | 0.81 (0.61 to 1.07) | - | - | - |
| HRS | 1,468 | 2,343 | 0.63 (0.55 to 0.72) | - | - | - |
| OPERAM | 234 | 418 | 0.73 (0.38 to 1.38) | - | - | - |
| SHARE | 4,294 | 8,307 | 0.93 (0.86 to 1.02) | - | - | - |

T2D & no CHD: participants with T2D but no established CHD at baseline; CHD & no T2D: participants with established CHD but no T2D at baseline. Study-specific hazard ratios were estimated using competing-risk flexible parametric survival models adjusted for age, gender, BMI, smoking, alcohol consumption, prior stroke, use of antihypertensive drugs, and use of cholesterol-lowering drugs. Overall hazard ratios were calculated using a random-effects meta-analysis.

CHD, coronary heart disease; CVD, cardiovascular disease; HR, hazard ratio; T2D, type 2 diabetes

# Figure S1 Participant flow diagram


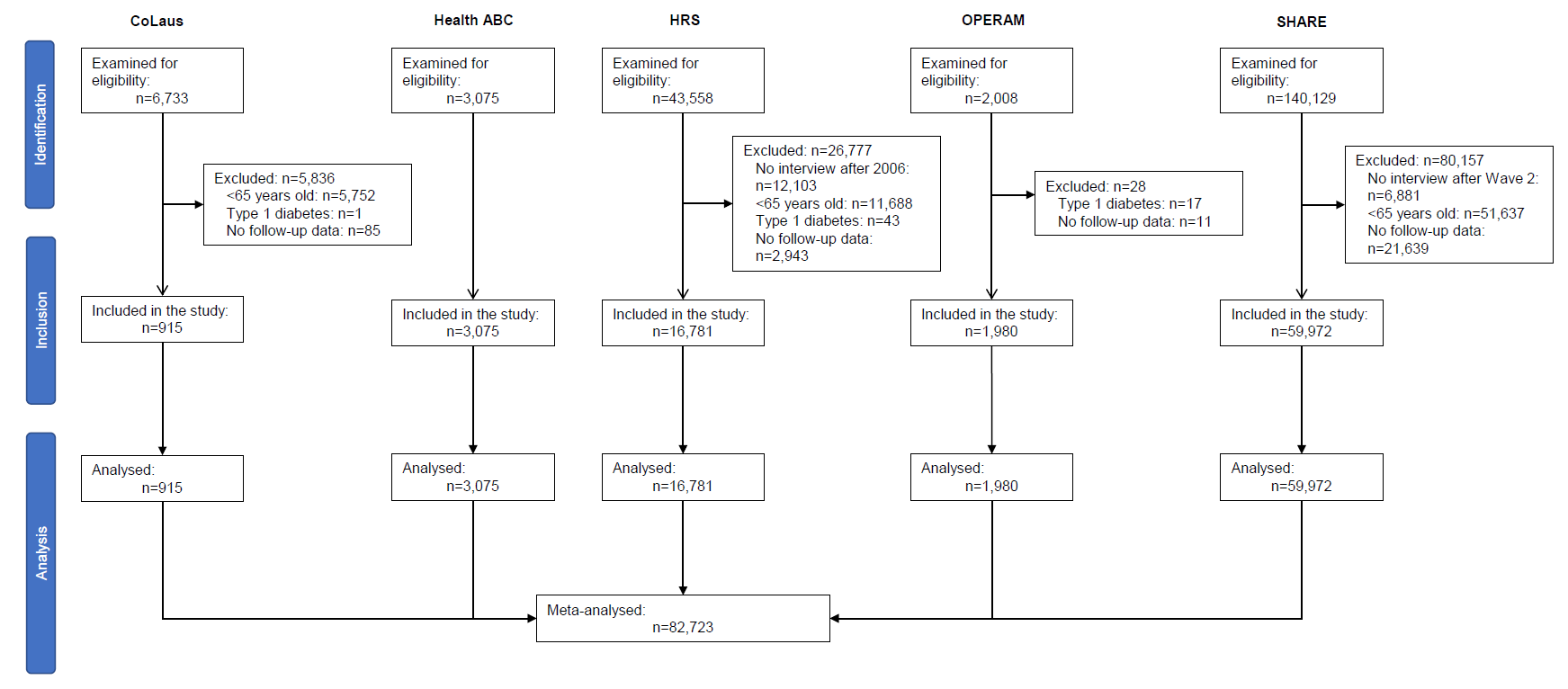


# Figure S2 Participant flow diagram (analyses on HbA1c)


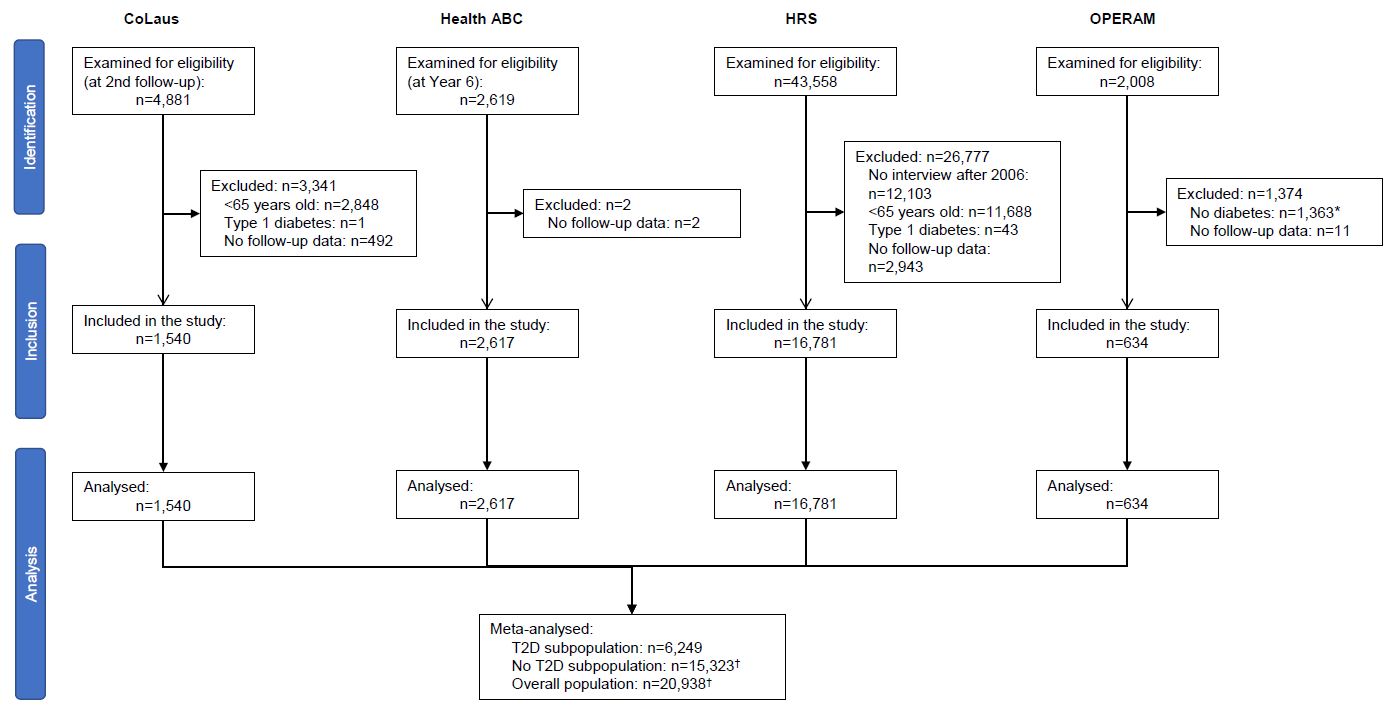


T2D, type 2 diabetes

* HbA1c was not collected for patients without diabetes in OPERAM

^†^ Excluding OPERAM, as HbA1c data was not collected in patients without diabetes

# Figure S3 Cumulative incidence curves by study and T2D status for composite outcome of CVD events or all-cause mortality


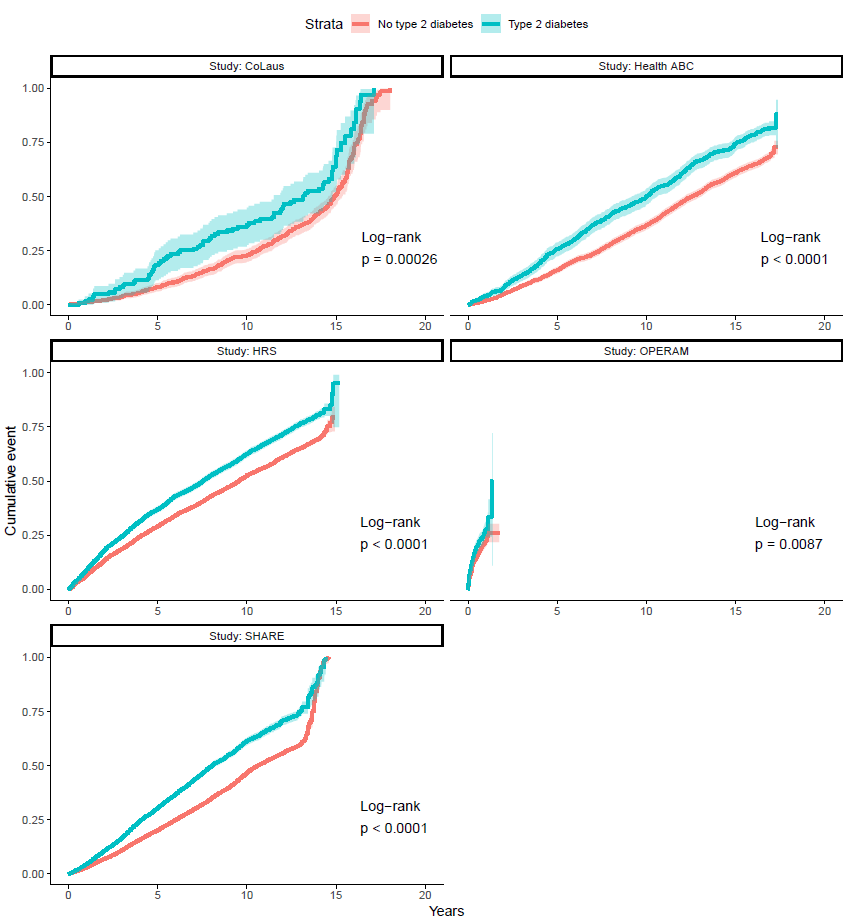


CVD, cardiovascular disease; T2D, type 2 diabetes

# Figure S4 Association between T2D and primary outcome of CVD events or all-cause mortality by study in individuals A) <75 years and B) ≥75 years of age


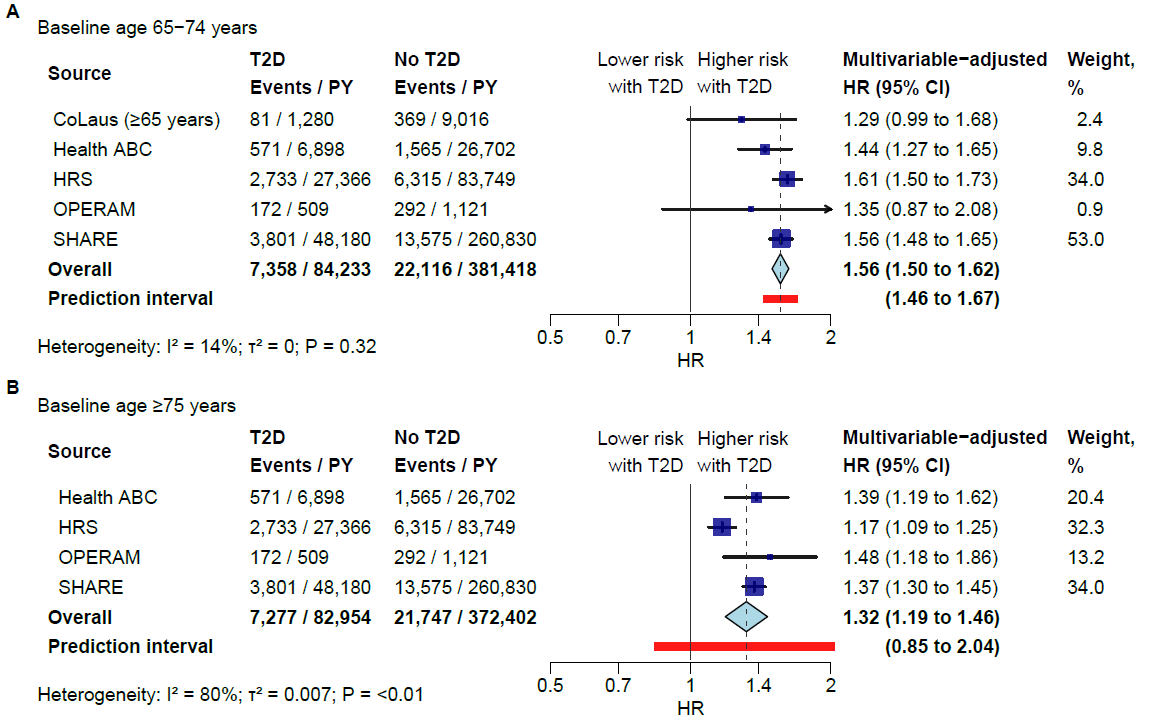


HR>1 indicates increased risk in T2D. Study-specific HRs for T2D were estimated for each age group using flexible parametric survival models adjusted for gender, BMI, smoking, alcohol consumption, prior CVD, use of antihypertensive drugs, and use of cholesterol-lowering drugs. Overall hazard ratios were calculated using a random-effects meta-analysis. No hazard ratios could be estimated for CoLaus in the age group ≥75 years as the maximum age in this cohort was 75 years.

CVD, cardiovascular disease; HR, hazard ratio; PY, person-years at risk; T2D, type 2 diabetes

# Figure S5 Association between T2D and outcomes using an age- and gender-adjusted model


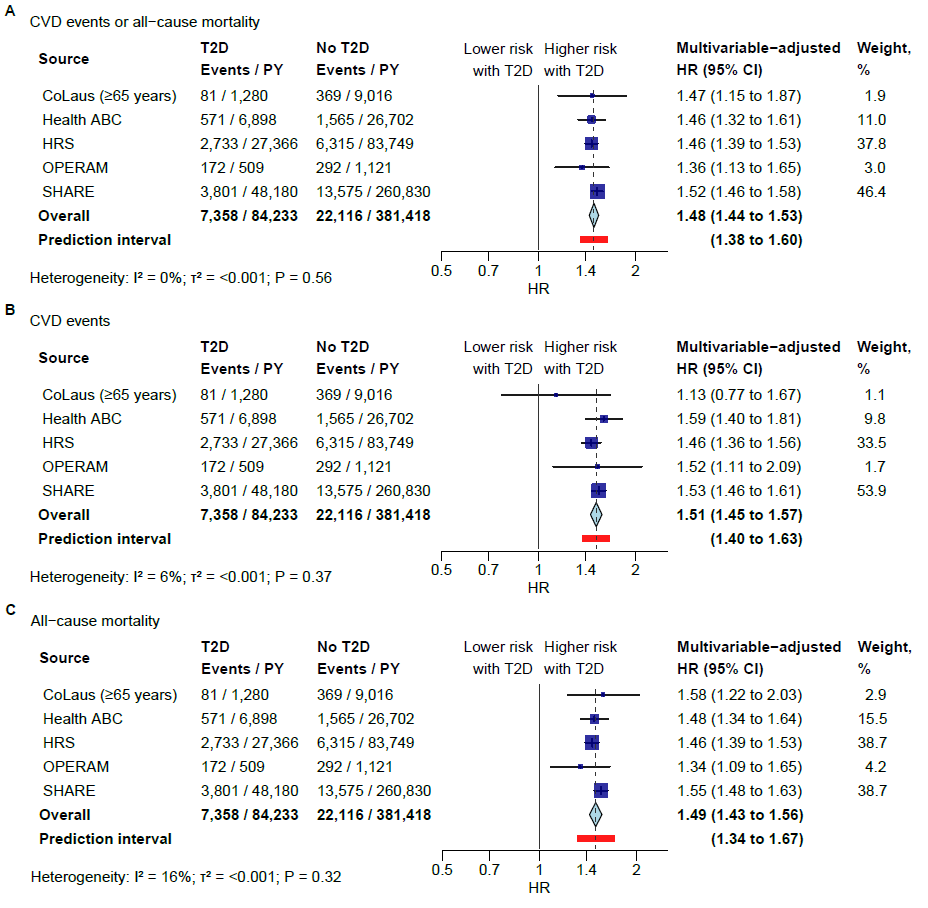


HR>1 indicates increased risk in T2D. Study-specific hazard ratios for T2D were estimated using flexible parametric survival models and combined using a random-effects meta-analysis.

CVD, cardiovascular disease; PY, person-years at risk; T2D, type 2 diabetes

# Figure S6 Association between T2D and outcomes using a fully adjusted model*


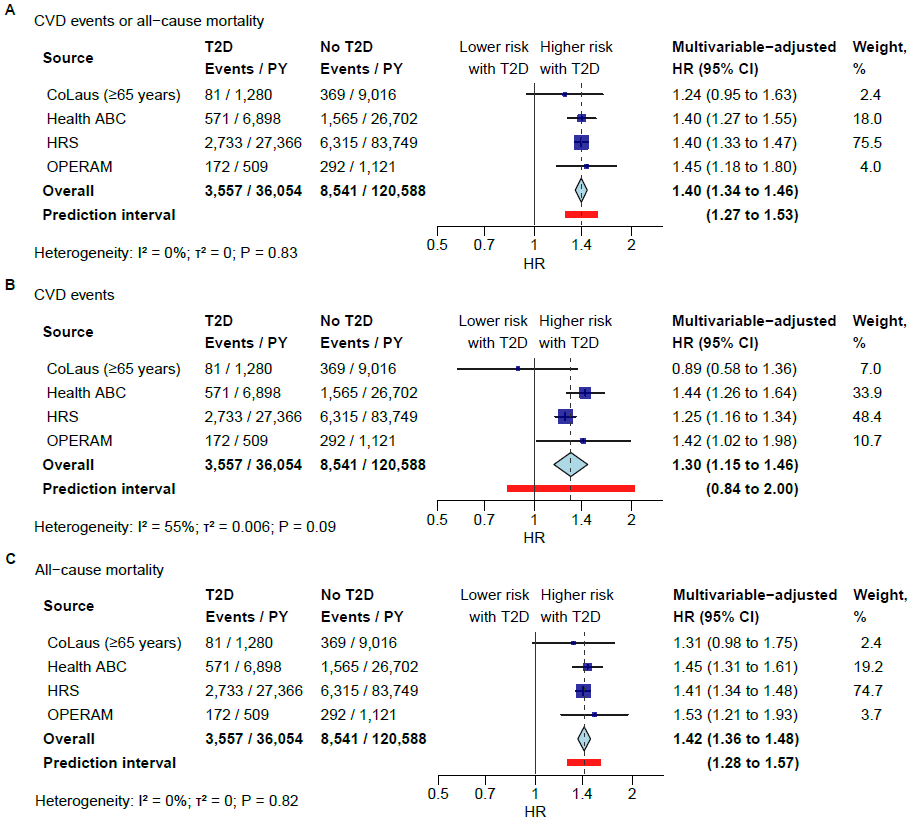


HR>1 indicates increased risk in T2D. Study-specific hazard ratios for T2D were estimated using flexible parametric survival models and combined using a random-effects meta-analysis.

* Models were adjusted for age, gender, BMI, smoking, alcohol consumption, prior CVD, use of antihypertensive drugs, use of cholesterol-lowering drugs, SBP, total cholesterol and HDL cholesterol.

CVD, cardiovascular disease; PY, person-years at risk; T2D, type 2 diabetes

# Figure S7 Association between T2D and secondary outcome of CVD events using a non-competing-risk flexible parametric survival model


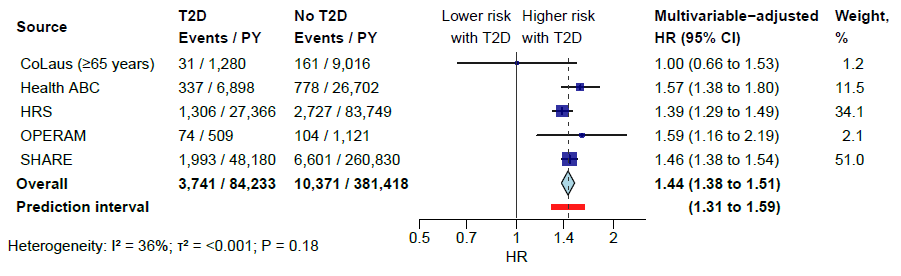


Study-specific hazard ratios for T2D were estimated using flexible parametric survival models adjusted for age, gender, BMI, smoking, alcohol consumption, prior CVD, use of antihypertensive drugs, and use of cholesterol-lowering drugs. Overall hazard ratios were calculated using a random-effects meta-analysis.

CVD, cardiovascular disease; PY, person-years at risk; T2D, type 2 diabetes

# Figure S8 Cumulative incidence curves by study and T2D and CHD status for composite outcome of CVD events or all-cause mortality


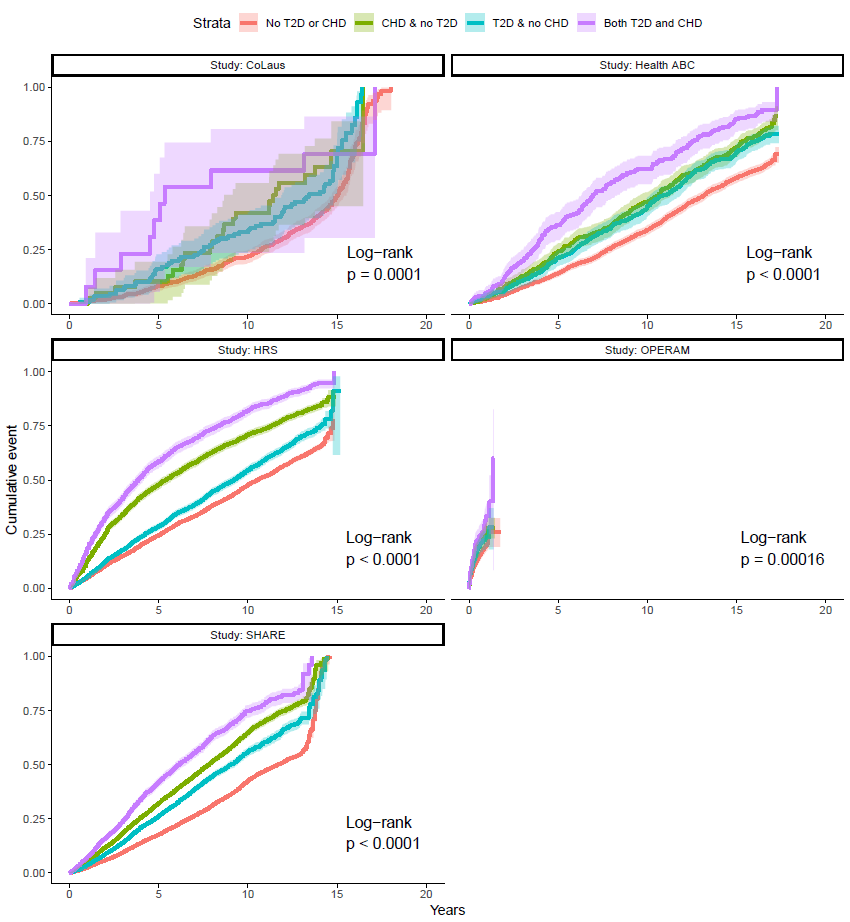


T2D & no CHD: participants with T2D but no established CHD at baseline; CHD & no T2D: participants with established CHD but no T2D at baseline.

CHD, coronary heart disease; CVD, cardiovascular disease; T2D, type 2 diabetes

# Figure S9 Association between T2D & no CHD vs CHD & no T2D and outcomes using an age- and gender-adjusted model


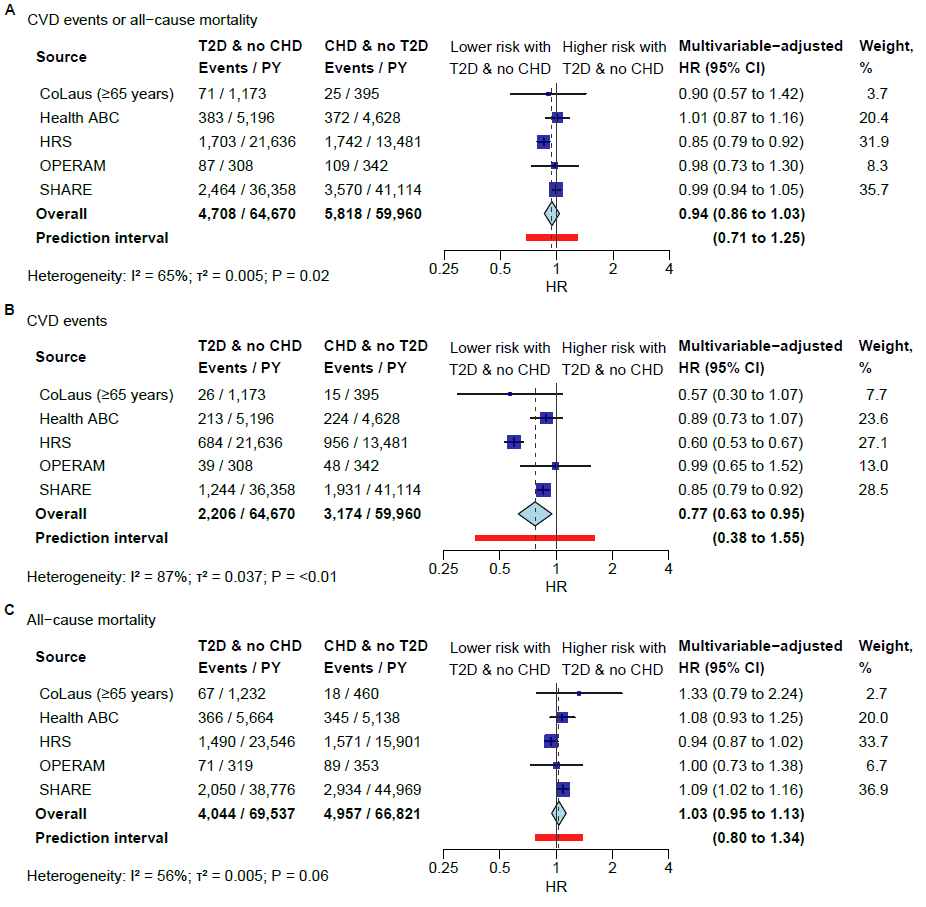


HR>1 indicates increased risk in individuals with T2D & no CHD. T2D & no CHD: participants with T2D but no established CHD at baseline; CHD & no T2D: participants with established CHD but no T2D at baseline. Study-specific hazard ratios for T2D were estimated using flexible parametric survival models and combined using a random-effects meta-analysis.

CHD, coronary heart disease; CVD, cardiovascular disease; PY, person-years at risk; T2D, type 2 diabetes

# Figure S10 Association between T2D & no CHD vs CHD & no T2D and outcomes using a fully adjusted model*


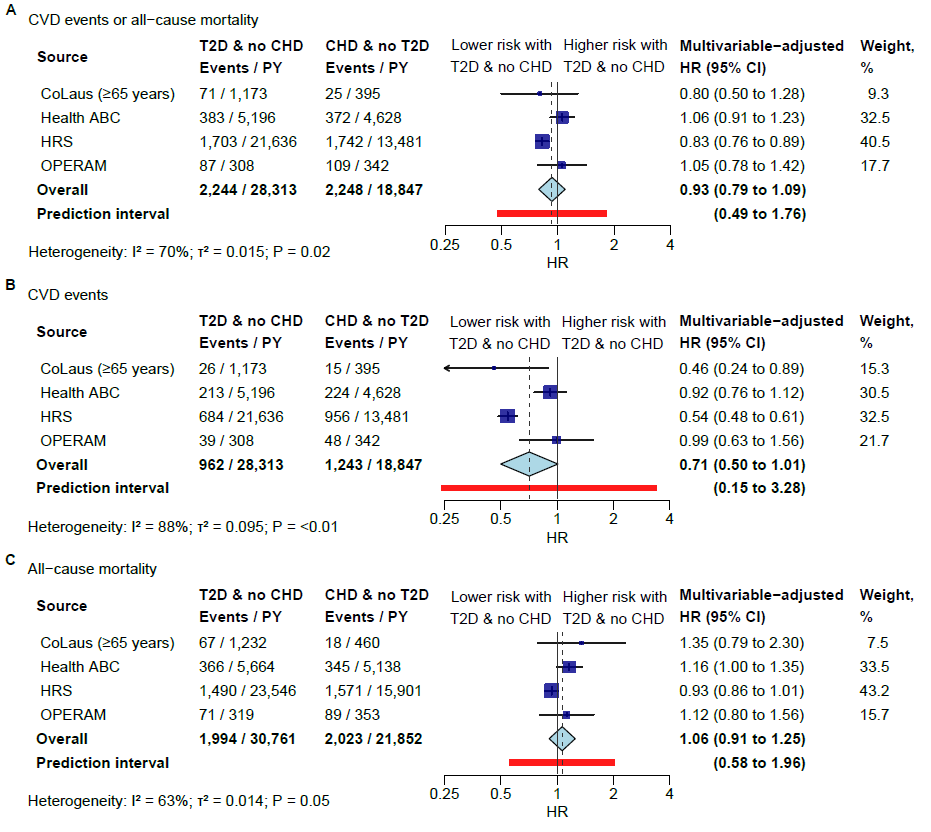


HR>1 indicates increased risk in individuals with T2D & no CHD. T2D & no CHD: participants with T2D but no established CHD at baseline; CHD & no T2D: participants with established CHD but no T2D at baseline. Study-specific hazard ratios for T2D were estimated using flexible parametric survival models and combined using a random-effects meta-analysis.

* Models were adjusted for age, gender, BMI, smoking, alcohol consumption, prior stroke, use of antihypertensive drugs, use of cholesterol-lowering drugs, SBP, total cholesterol and HDL cholesterol.

CHD, coronary heart disease; CVD, cardiovascular disease; PY, person-years at risk; T2D, type 2 diabetes

# Figure S11 Association between T2D & no CHD vs CHD & no T2D and CVD events using a non-competing-risk flexible parametric survival model


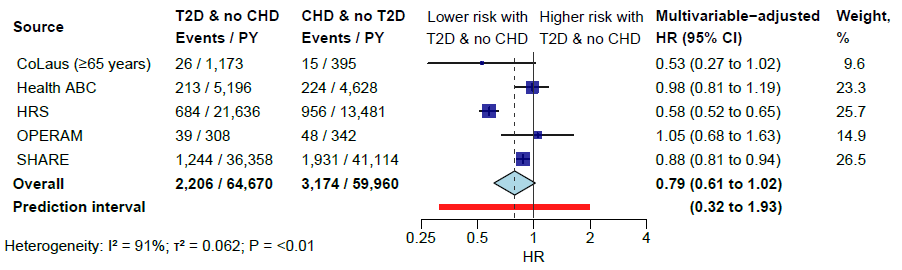


Study-specific hazard ratios for T2D were estimated using flexible parametric survival models adjusted for age, gender, BMI, smoking, alcohol consumption, prior stroke, use of antihypertensive drugs, and use of cholesterol-lowering drugs. Overall hazard ratios were calculated using a random-effects meta-analysis.

CHD, coronary heart disease, CVD, cardiovascular disease; PY, person-years at risk; T2D, type 2 diabetes

# Figure S12 Association between T2D & no CVD* vs CVD* & no T2D and outcomes


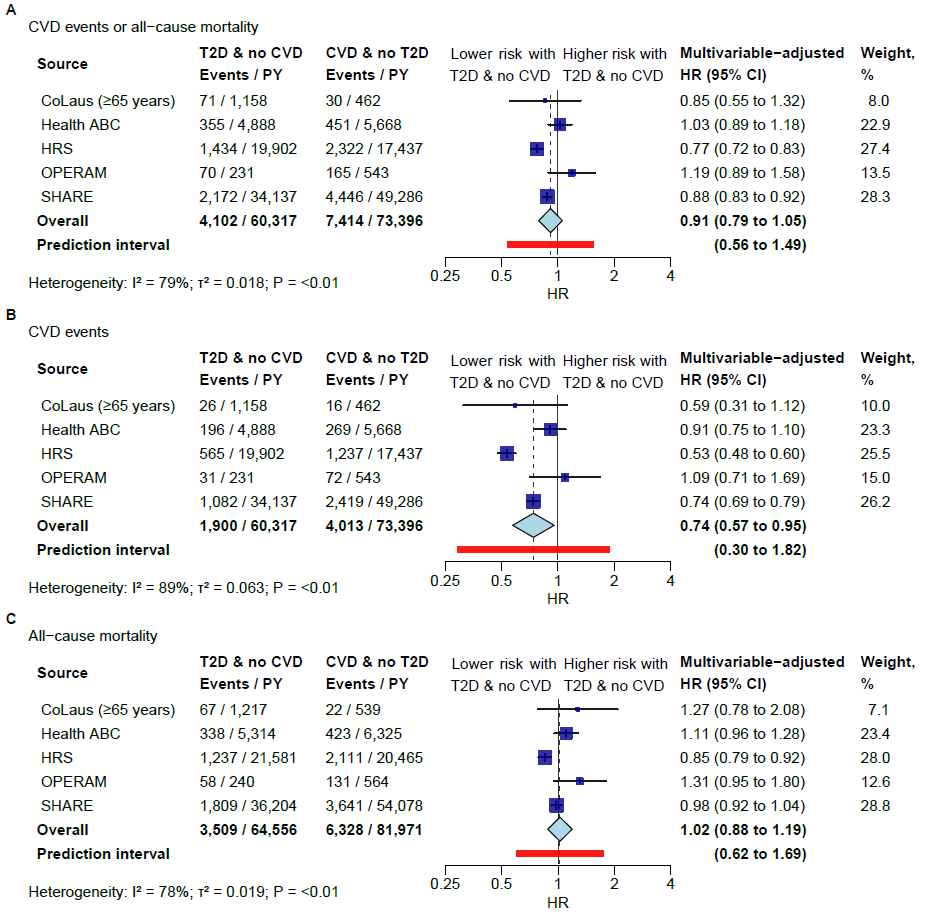


HR>1 indicates increased risk in individuals with T2D & no CVD. T2D & no CVD: participants with T2D but no established CVD (defined as history of CHD or stroke) at baseline; CVD & no T2D: participants with established CVD (defined as history of CHD or stroke) but no T2D at baseline. Study-specific hazard ratios for T2D were estimated using flexible parametric survival models and combined using a random-effects meta-analysis.

CVD, cardiovascular disease; PY, person-years at risk; T2D, type 2 diabetes

# Figure S13 Subgroup analyses of the association between HbA1c and the composite outcome of CVD events or all-cause mortality in the T2D subpopulation


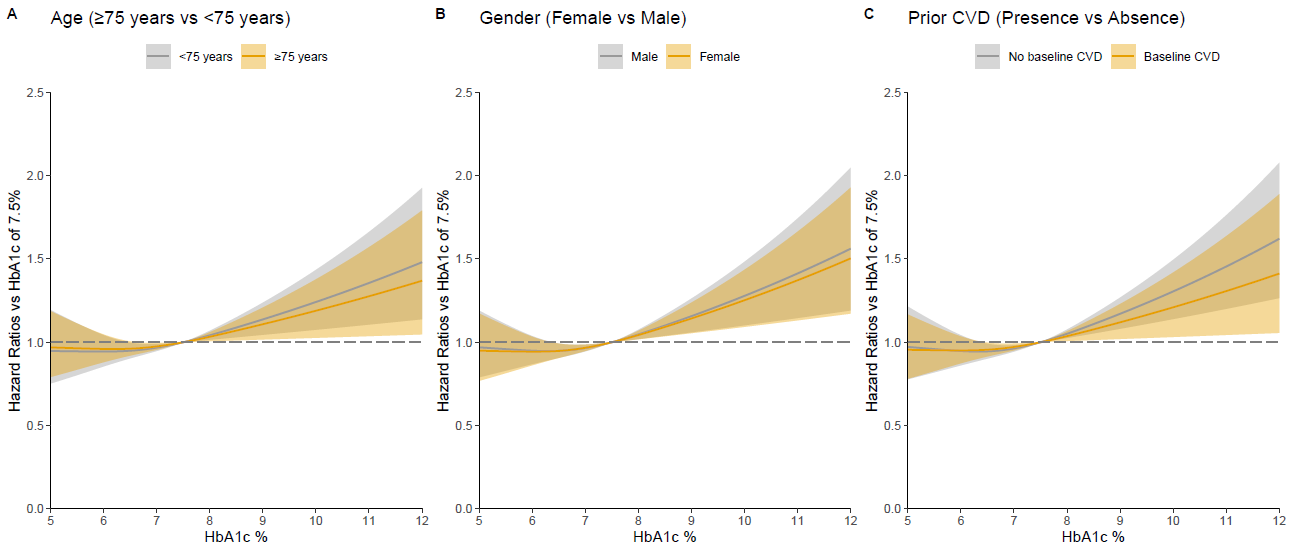


Subgroup hazard ratios for HbA1c were estimated for each study by using flexible parametric survival models in which interaction terms of HbA1c and each subgroup were included. Models were adjusted for age, gender, BMI, smoking, alcohol consumption, prior CVD, use of antihypertensive drugs, and use of cholesterol-lowering drugs. HbA1c was modelled as a continuous variable using splines with three knots at the 10^th^, 50^th^ and 90^th^ percentile. Spline coefficients were combined using a multivariate random-effects meta-analysis model and displayed in these line graphs. Hazard ratios and confidence intervals were calculated in reference to an HbA1c value of 7.5%.

CVD, cardiovascular disease; HbA1c, haemoglobin A1c; T2D, type 2 diabetes

# Figure S14 Age subgroup analyses of the association between HbA1c and the CVD events outcome in the T2D subpopulation


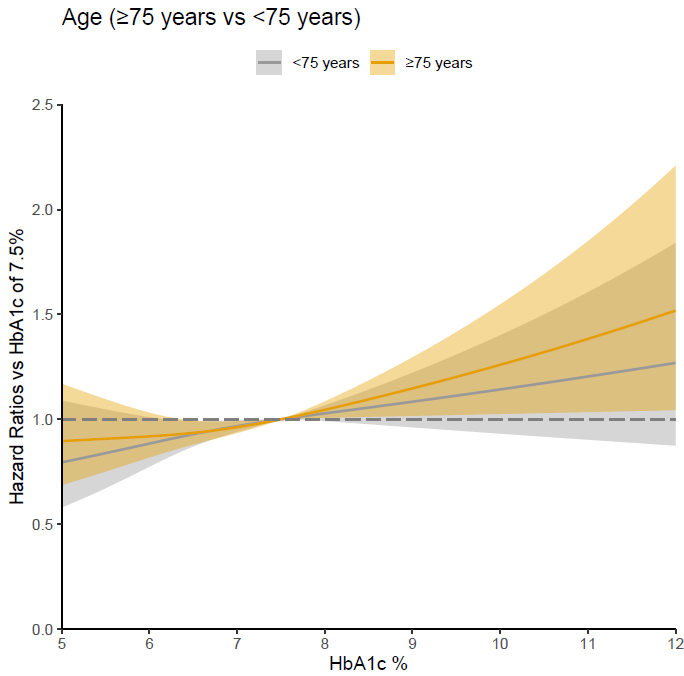


The age subgroup hazard ratios for HbA1c were estimated for each study (except OPERAM, due to issues with model convergence) by using flexible parametric survival models in which interaction terms of HbA1c and each subgroup were included. Models were adjusted for age, gender, BMI, smoking, alcohol consumption, prior CVD, use of antihypertensive drugs, and use of cholesterol-lowering drugs. HbA1c was modelled as a continuous variable using splines with three knots at the 10^th^, 50^th^ and 90^th^ percentile. Spline coefficients were combined using a multivariate random-effects meta-analysis model and displayed in these line graphs. Hazard ratios and confidence intervals were calculated in reference to an HbA1c value of 7.5%.

CVD, cardiovascular disease; HbA1c, haemoglobin A1c; T2D, type 2 diabetes

# Figure S15 Association between continuous HbA1c and CVD events or all-cause mortality in the T2D subpopulation; excluding patients using insulin, sulfonylureas or glinides


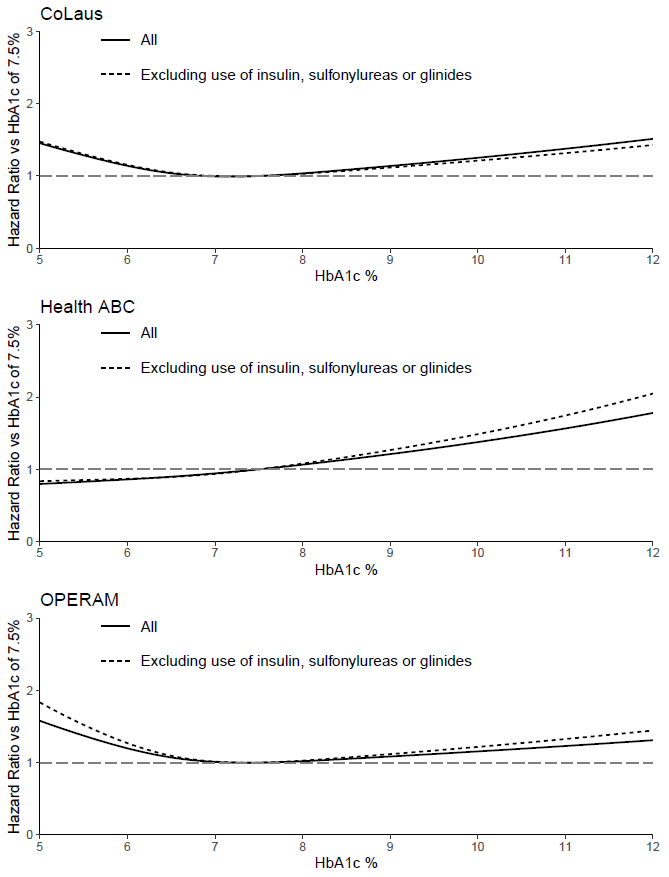


Hazard ratios for HbA1c were estimated for each study by using flexible parametric survival models adjusted for age, gender, BMI, smoking, alcohol consumption, prior CVD, use of antihypertensive drugs, and use of cholesterol-lowering drugs. Models were performed once with all participants included and once excluding participants that used insulin, sulfonylureas or glinides. HbA1c was modelled as a continuous variable using splines with three knots at the 10^th^, 50^th^ and 90^th^ percentile. Hazard ratios and confidence intervals were calculated in reference to an HbA1c value of 7.5%. HRS is not included in this analysis as information on the types of antidiabetic drug was not available.

CVD, cardiovascular disease; HbA1c, hemoglobin A1c; T2D, type 2 diabetes

# Figure S16 Association between HbA1c and CVD events using a non-competing-risk flexible parametric survival model


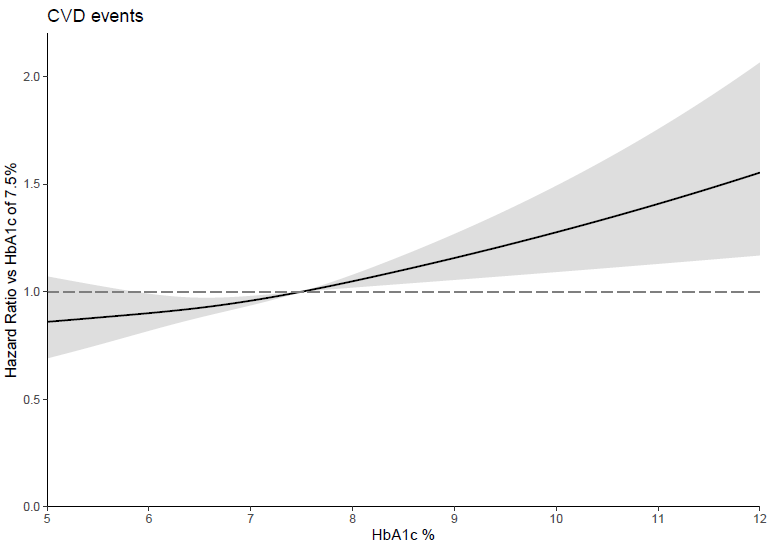


Hazard ratios for HbA1c were estimated for each study by using flexible parametric survival models adjusted for age, gender, BMI, smoking, alcohol consumption, prior CVD, use of antihypertensive drugs, and use of cholesterol-lowering drugs. HbA1c was modelled as a continuous variable using splines with three knots at the 10^th^, 50^th^ and 90^th^ percentile. Spline coefficients were combined using a multivariate random-effects meta-analysis model and displayed in the line graph. Hazard ratios and confidence intervals were calculated in reference to an HbA1c value of 7.5%.

CVD, cardiovascular disease; HbA1c, haemoglobin A1c; T2D, type 2 diabetes

# Figure S17 Association between HbA1c and outcomes in older adults without T2D


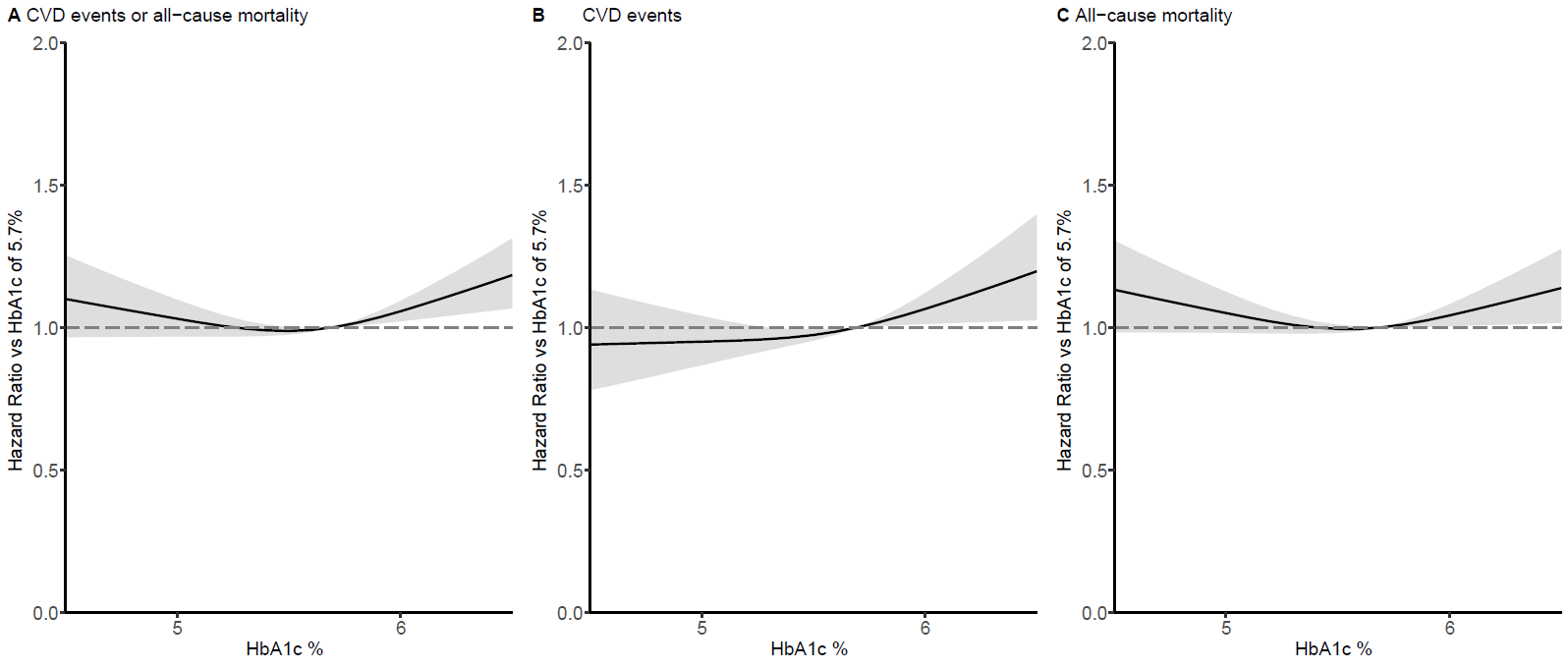


Hazard ratios for HbA1c were estimated for each study by using flexible parametric survival models adjusted for age, gender, BMI, smoking, alcohol consumption, prior CVD, use of antihypertensive drugs, and use of cholesterol-lowering drugs. A competing-risk-adjusted model was used for the secondary outcome of CVD events (Panel B). HbA1c was modelled as a continuous variable using splines with three knots at the 10^th^, 50^th^ and 90^th^ percentile. Spline coefficients were combined using a multivariate random-effects meta-analysis model and displayed in these line graphs. Hazard ratios and confidence intervals were calculated in reference to an HbA1c value of 5.7%.

CVD, cardiovascular disease; HbA1c, haemoglobin A1c; T2D, type 2 diabetes

# Figure S18 Association between HbA1c and outcomes in older adults with or without T2D


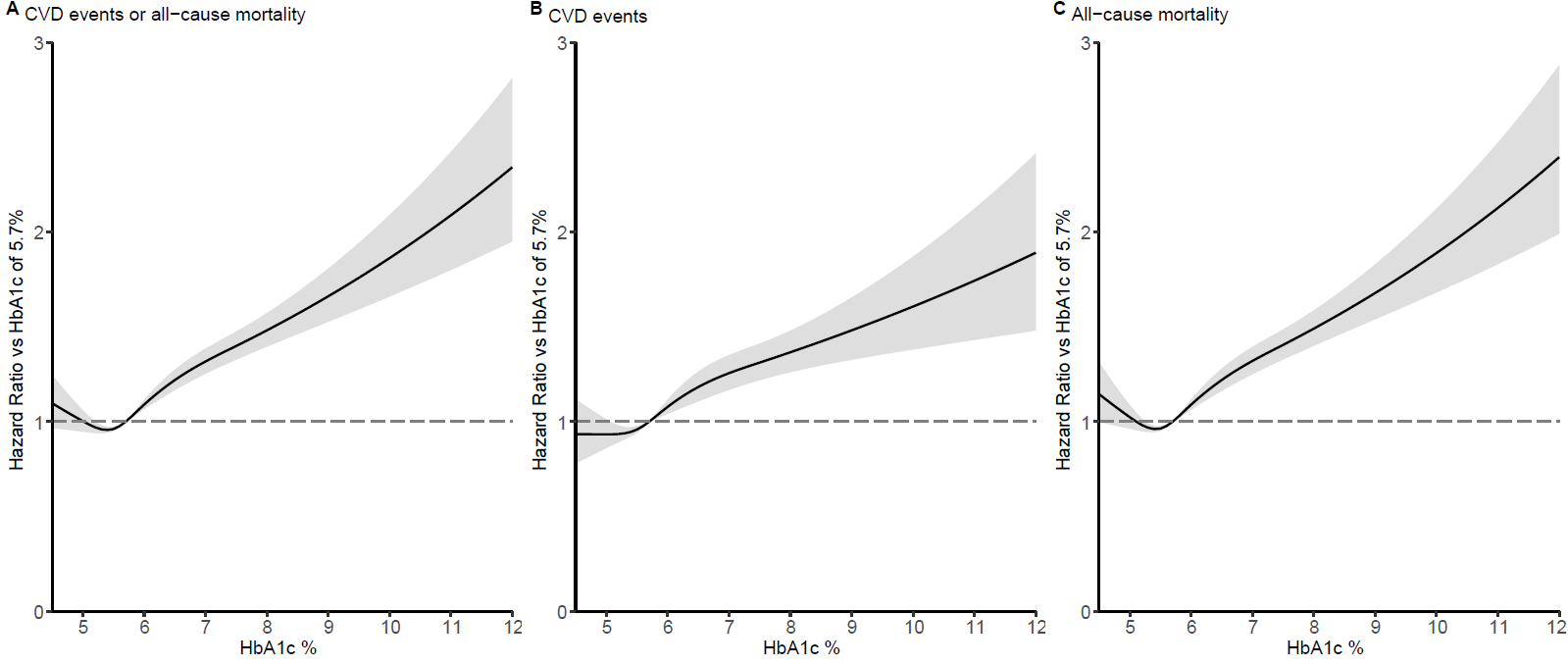


Hazard ratios for HbA1c were estimated for each study by using flexible parametric survival models adjusted for age, gender, BMI, smoking, alcohol consumption, prior CVD, use of antihypertensive drugs, and use of cholesterol-lowering drugs. A competing-risk-adjusted model was used for the secondary outcome of CVD events (Panel B). HbA1c was modelled as a continuous variable using splines with four knots at the 5^th^, 35^th^ 65^th^ and 95^th^ percentile. Spline coefficients were combined using a multivariate random-effects meta-analysis model and displayed in these line graphs. Hazard ratios and confidence intervals were calculated in reference to an HbA1c value of 5.7%.

CVD, cardiovascular disease; HbA1c, haemoglobin A1c; T2D, type 2 diabetes

# References

1. Elm Ev, Altman DG, Egger M, Pocock SJ, Gøtzsche PC, Vandenbroucke JP. Strengthening the reporting of observational studies in epidemiology (STROBE) statement: guidelines for reporting observational studies. BMJ 2007;335(7624):806-808. doi:10.1136/bmj.39335.541782.AD

2. Firmann M, Mayor V, Vidal PM, et al. The CoLaus study: a population-based study to investigate the epidemiology and genetic determinants of cardiovascular risk factors and metabolic syndrome. BMC Cardiovasc Disord 2008;8:6. doi:10.1186/1471-2261-8-6

3. Rodondi N, Marques-Vidal P, Butler J, et al. Markers of atherosclerosis and inflammation for prediction of coronary heart disease in older adults. Am J Epidemiol 2010;171(5):540-9. doi:10.1093/aje/kwp428

4. Sonnega A, Faul JD, Ofstedal MB, Langa KM, Phillips JW, Weir DR. Cohort Profile: the Health and Retirement Study (HRS). Int J Epidemiol 2014;43(2):576-85. doi:10.1093/ije/dyu067

5. Health and Retirement Study. (2020 HRS Core) public use dataset. Produced and distributed by the University of Michigan with funding from the National Institute on Aging (grant number NIA U01AG009740).

6. Health and Retirement Study. (2018 HRS Exit) public use dataset. Produced and distributed by the University of Michigan with funding from the National Institute on Aging (grant number NIA U01AG009740).

7. Health and Retirement Study. (2018 HRS Core) public use dataset. Produced and distributed by the University of Michigan with funding from the National Institute on Aging (grant number NIA U01AG009740).

8. Health and Retirement Study. (2016 Venous Blood Study (VBS)) public use dataset. Produced and distributed by the University of Michigan with funding from the National Institute on Aging (grant number NIA U01AG009740).

9. Health and Retirement Study. (2016 HRS Exit) public use dataset. Produced and distributed by the University of Michigan with funding from the National Institute on Aging (grant number NIA U01AG009740).

10. Health and Retirement Study. (2016 HRS Core) public use dataset. Produced and distributed by the University of Michigan with funding from the National Institute on Aging (grant number NIA U01AG009740).

11. Health and Retirement Study. (2016 Biomarker Data) public use dataset. Produced and distributed by the University of Michigan with funding from the National Institute on Aging (grant number NIA U01AG009740).

12. Health and Retirement Study. (2014 HRS Exit) public use dataset. Produced and distributed by the University of Michigan with funding from the National Institute on Aging (grant number NIA U01AG009740).

13. Health and Retirement Study. (2014 HRS Core) public use dataset. Produced and distributed by the University of Michigan with funding from the National Institute on Aging (grant number NIA U01AG009740).

14. Health and Retirement Study. (2014 Biomarker Data) public use dataset. Produced and distributed by the University of Michigan with funding from the National Institute on Aging (grant number NIA U01AG009740).

15. Health and Retirement Study. (2012 HRS Exit) public use dataset. Produced and distributed by the University of Michigan with funding from the National Institute on Aging (grant number NIA U01AG009740).

16. Health and Retirement Study. (2012 HRS Core) public use dataset. Produced and distributed by the University of Michigan with funding from the National Institute on Aging (grant number NIA U01AG009740).

17. Health and Retirement Study. (2012 Biomarker Data) public use dataset. Produced and distributed by the University of Michigan with funding from the National Institute on Aging (grant number NIA U01AG009740).

18. Health and Retirement Study. (2010 HRS Exit) public use dataset. Produced and distributed by the University of Michigan with funding from the National Institute on Aging (grant number NIA U01AG009740).

19. Health and Retirement Study. (2010 HRS Core) public use dataset. Produced and distributed by the University of Michigan with funding from the National Institute on Aging (grant number NIA U01AG009740).

20. Health and Retirement Study. (2010 Biomarker Data) public use dataset. Produced and distributed by the University of Michigan with funding from the National Institute on Aging (grant number NIA U01AG009740).

21. Health and Retirement Study. (2008 HRS Exit) public use dataset. Produced and distributed by the University of Michigan with funding from the National Institute on Aging (grant number NIA U01AG009740).

22. Health and Retirement Study. (2008 HRS Core) public use dataset. Produced and distributed by the University of Michigan with funding from the National Institute on Aging (grant number NIA U01AG009740).

23. Health and Retirement Study. (2008 Biomarker Data) public use dataset. Produced and distributed by the University of Michigan with funding from the National Institute on Aging (grant number NIA U01AG009740).

24. Health and Retirement Study. (2006 HRS Exit) public use dataset. Produced and distributed by the University of Michigan with funding from the National Institute on Aging (grant number NIA U01AG009740).

25. Health and Retirement Study. (2006 HRS Core) public use dataset. Produced and distributed by the University of Michigan with funding from the National Institute on Aging (grant number NIA U01AG009740).

26. Health and Retirement Study. (2006 Biomarker Data) public use dataset. Produced and distributed by the University of Michigan with funding from the National Institute on Aging (grant number NIA U01AG009740).

27. Blum MR, Sallevelt BTGM, Spinewine A, et al. Optimizing Therapy to Prevent Avoidable Hospital Admissions in Multimorbid Older Adults (OPERAM): cluster randomised controlled trial. BMJ 2021;374:n1585. doi:10.1136/bmj.n1585

28. Börsch-Supan A, Brandt M, Hunkler C, et al. Data Resource Profile: the Survey of Health, Ageing and Retirement in Europe (SHARE). Int J Epidemiol 2013;42(4):992-1001. doi:10.1093/ije/dyt088

29. Bergmann M, Kneip T, Luca GD, Scherpenzeel A. Survey participation in the Survey of Health, Ageing and Retirement in Europe (SHARE), Wave 1-7. Based on Release 7.0.0. SHARE Working Paper Series 41-2019. 2019.

30. Börsch-Supan A, Brugiavini A, Jürges H, et al, eds. First results from the Survey of Health, Ageing and Retirement in Europe (2004-2007). Starting the longitudinal dimension. Mannheim Research Institute for the Economics of Aging (MEA); 2008.

31. Bergmann M, Börsch-Supan A, eds. SHARE Wave 8 Methodology: Collecting Cross-National Survey Data in Times of COVID-19. MEA, Max Planck Institute for Social Law and Social Policy; 2021.

32. Bergmann M, Scherpenzeel A, Börsch-Supan A, eds. SHARE Wave 7 Methodology: Panel Innovations and Life Histories. MEA, Max Planck Institute for Social Law and Social Policy; 2019.

33. Malter F, Börsch-Supan A, eds. SHARE Wave 6: Panel innovations and collecting Dried Blood Spots. MEA, Max Planck Institute for Social Law and Social Policy; 2017.

34. Malter F, Börsch-Supan A, eds. SHARE Wave 5: Innovations & Methodology. MEA, Max Planck Institute for Social Law and Social Policy; 2015.

35. Malter F, Börsch-Supan A, eds. SHARE Wave 4: Innovations & Methodology. MEA, Max Planck Institute for Social Law and Social Policy; 2013.

36. Börsch-Supan A. Survey of Health, Ageing and Retirement in Europe (SHARE) Wave 2. Release version: 8.0.0. SHARE-ERIC. Data set. 2022. doi:10.6103/SHARE.w2.800

37. Börsch-Supan A. Survey of Health, Ageing and Retirement in Europe (SHARE) Wave 4. Release version: 8.0.0. SHARE-ERIC. Data set. 2022. doi:10.6103/SHARE.w4.800

38. Börsch-Supan A. Survey of Health, Ageing and Retirement in Europe (SHARE) Wave 5. Release version: 8.0.0. SHARE-ERIC. Data set. 2022. doi:10.6103/SHARE.w5.800

39. Börsch-Supan A. Survey of Health, Ageing and Retirement in Europe (SHARE) Wave 6. Release version: 8.0.0. SHARE-ERIC. Data set. 2022. doi:10.6103/SHARE.w6.800

40. Börsch-Supan A. Survey of Health, Ageing and Retirement in Europe (SHARE) Wave 7. Release version: 8.0.0. SHARE-ERIC. Data set. 2022. doi:10.6103/SHARE.w7.800

41. Börsch-Supan A. Survey of Health, Ageing and Retirement in Europe (SHARE) Wave 8. Release version: 8.0.0. SHARE-ERIC. Data set. 2022. doi:10.6103/SHARE.w8.800

42. Aponte Ribero V, Alwan H, Efthimiou O, et al. Cardiovascular disease and type 2 diabetes in older adults: a combined protocol for an individual participant data analysis for risk prediction and a network meta-analysis of novel anti-diabetic drugs. medRxiv 2023;doi:10.1101/2023.03.13.23287105

43. Lambert PC. The estimation and modelling of cause-specific cumulative incidence functions using time-dependent weights. Stata J 2017;17(1):181-207.

44. Jackson D, Riley R, White IR. Multivariate meta-analysis: potential and promise. Stat Med 2011;30(20):2481-98. doi:10.1002/sim.4172

45. Draznin B, Aroda VR, Bakris G, et al. 13. Older Adults: Standards of Medical Care in Diabetes-2022. Diabetes Care 2022;45(Suppl 1):S195-s207. doi:10.2337/dc22-S013

46. American Diabetes Association. 2. Classification and Diagnosis of Diabetes: Standards of Medical Care in Diabetes—2021. Diabetes Care 2020;44(Supplement_1):S15-S33. doi:10.2337/dc21-S002

47. LeRoith D, Biessels GJ, Braithwaite SS, et al. Treatment of Diabetes in Older Adults: An Endocrine Society* Clinical Practice Guideline. J Clin Endocrinol Metab 2019;104(5):1520-1574. doi:10.1210/jc.2019-00198

48. Zhao Y, Malik S, Budoff MJ, et al. Identification and Predictors for Cardiovascular Disease Risk Equivalents among Adults With Diabetes Mellitus. Diabetes Care 2021;doi:10.2337/dc21-0431

49. *R: A language and environment for statistical computing*. R Foundation for Statistical Computing, Vienna, Austria; 2021. <https://www.R-project.org/>

50. van Buuren S, Groothuis-Oudshoorn K. mice: Multivariate Imputation by Chained Equations in R. Journal of Statistical Software 2011;45(3):1 - 67. doi:10.18637/jss.v045.i03

51. Jackson C. flexsurv: A Platform for Parametric Survival Modeling in R. Journal of Statistical Software 2016;70(8):1 - 33. doi:10.18637/jss.v070.i08

52. Balduzzi S, Rücker G, Schwarzer G. How to perform a meta-analysis with R: a practical tutorial. Evid Based Ment Health 2019;22(4):153-160. doi:10.1136/ebmental-2019-300117

53. Gasparrini A, Armstrong B, Kenward MG. Multivariate meta-analysis for non-linear and other multi-parameter associations. Stat Med 2012;31(29):3821-39. doi:10.1002/sim.5471

54. Kalinowski A, Humphreys K. Governmental standard drink definitions and low-risk alcohol consumption guidelines in 37 countries. Addiction 2016;111(7):1293-8. doi:10.1111/add.13341
